# Differentially expressed genes linked to natural variation in long-term memory formation in *Cotesia* parasitic wasps

**DOI:** 10.3389/fnbeh.2015.00255

**Published:** 2015-09-25

**Authors:** Joke J. F. A. van Vugt, Katja M. Hoedjes, Henri C. van de Geest, Elio W. G. M. Schijlen, Louise E. M. Vet, Hans M. Smid

**Affiliations:** ^1^Department of Terrestrial Ecology, Netherlands Institute of Ecology (NIOO-KNAW)Wageningen, Netherlands; ^2^Laboratory of Entomology, Wageningen UniversityWageningen, Netherlands; ^3^Applied Bioinformatics, Plant Research InternationalWageningen, Netherlands

**Keywords:** Cotesia glomerata, Cotesia rubecula, parasitic wasp, strand-specific RNAseq, long-term memory, anesthesia-resistant memory, differential expression analysis

## Abstract

Even though learning and memory are universal traits in the Animal Kingdom, closely related species reveal substantial variation in learning rate and memory dynamics. To determine the genetic background of this natural variation, we studied two congeneric parasitic wasp species, *Cotesia glomerata* and *C. rubecula*, which lay their eggs in caterpillars of the large and small cabbage white butterfly. A successful egg laying event serves as an unconditioned stimulus (US) in a classical conditioning paradigm, where plant odors become associated with the encounter of a suitable host caterpillar. Depending on the host species, the number of conditioning trials and the parasitic wasp species, three different types of transcription-dependent long-term memory (LTM) and one type of transcription-independent, anesthesia-resistant memory (ARM) can be distinguished. To identify transcripts underlying these differences in memory formation, we isolated mRNA from parasitic wasp heads at three different time points between induction and consolidation of each of the four memory types, and for each sample three biological replicates, where after strand-specific paired-end 100 bp deep sequencing. Transcriptomes were assembled *de novo* and differential expression was determined for each memory type and time point after conditioning, compared to unconditioned wasps. Most differentially expressed (DE) genes and antisense transcripts were only DE in one of the LTM types. Among the DE genes that were DE in two or more LTM types, were many protein kinases and phosphatases, small GTPases, receptors and ion channels. Some genes were DE in opposing directions between any of the LTM memory types and ARM, suggesting that ARM in *Cotesia* requires the transcription of genes inhibiting LTM or* vice versa*. We discuss our findings in the context of neuronal functioning, including RNA splicing and transport, epigenetic regulation, neurotransmitter/peptide synthesis and antisense transcription. In conclusion, these brain transcriptomes provide candidate genes that may be involved in the observed natural variation in LTM in closely related *Cotesia* parasitic wasp species.

## Introduction

Recent insights into the homology between brains of invertebrates and vertebrates suggest that a common ancestor’s bilatarian brain already possessed the ground patterns required for complex tasks such as learning and memory (Strausfeld and Hirth, 2013). Indeed, learning and memory are universal traits in the Animal Kingdom, and underlying mechanisms are remarkably similar, both at the level of the behavioral properties required for learning as well as the level of underlying genes required for memory formation (Dubnau, 2003). Whereas learning has been demonstrated in a wide array of insect species, two main model species are mostly studied, the honeybee *Apis mellifera* and the fruit fly *Drosophila melanogaster*.

In both species, associative learning events, like aversive and appetitive olfactory conditioning, induce formation of different forms of memory, which can be classified in three categories according to their sensitivity to disruptive treatments (Eisenhardt, 2006; Stough et al., 2006). Early memory like short-term memory (STM) can be erased by anesthesia, such as a cold-shock, and is therefore also named anesthesia-sensitive memory (ASM). More robust, longer lasting forms of memory are resistant to anesthesia, and hence called anesthesia-resistant memory (ARM). Long-term memory (LTM) consolidation requires protein synthesis, which is not the case for ARM. Thus, a distinction between LTM and ARM can be made by using translation or transcription inhibitors that inhibit LTM and not ARM. The idea emerging from many studies is that STM, ARM and LTM are independent memories that can occur in parallel, in different neurons (Blum and Dubnau, 2010), whereas some studies in *D. melanogaster* suggest that ARM and LTM are mutually exclusive (Isabel et al., 2004; Plaçais et al., 2012). The formation of specific forms of memory is triggered by the type and frequency of the conditioning trials. Usually, a single conditioning trial, or several trials with short inter-trial intervals in the range of seconds (massed conditioning), will induce STM and ARM but not LTM. Only spaced conditioning, i.e., multiple conditioning trials with intervals of several minutes, results in the formation of LTM, but there are exceptions; for instance a single appetitive food conditioning trial in *D. melanogaster* results in LTM (Krashes and Waddell, 2008).

An important aspect of memory dynamics is forgetting. This process has traditionally been interpreted as a passive decay process. However, the decay of memory, both with and without any interfering learning events (e.g., memory extinction, experiencing a learned cue without the expected reinforcer) or by retroactive interference (conflicting experiences) are caused by active dopaminergic signaling, and the activity of cytoskeleton remodelers (Berry and Davis, 2014). Thus, the result of single or multiple learning experiences results in the activation of diverse mechanisms that together determine the outcome; building a stable LTM or more transient forms of memory like STM or ARM.

Whereas the use of the traditional models has brought a wealth of insight in the mechanism of learning and memory, the aspect of natural variation has received little attention. We recently showed that profound variation in memory dynamics exists between closely related species of parasitic wasps. These wasps lay their eggs in host insects, and learn to associate cues, for instance odors, with a rewarding host encounter (Vet et al., 1995; Hoedjes et al., 2011). Like appetitive conditioning in *D. melanogaster*, some species form LTM after a single conditioning trial, but other species require multiple host encounters spaced in time. For instance, the parasitic wasp species *Nasonia* *vitripennis* forms transcription-dependent LTM for odors after a single encounter with its host, a fly pupa, whereas *N. giraulti* only forms ARM and requires multiple spaced experiences to form LTM (Hoedjes et al., 2012; Hoedjes and Smid, 2014).

Another comparison, and the focus of this study, is between the two species *Cotesia glomerata* and *C. rubecula. C. glomerata* parasitizes *Pieridae* caterpillars and can be conditioned using a classical conditioning assay in the lab, where plant odors induced by feeding of the caterpillars are the conditioned stimulus (CS) and the caterpillar host, including its excretions and produced silk form the unconditioned stimulus (US; Bleeker et al., 2006). It forms LTM after a single or three massed conditionings when its preferred host species, the large cabbage white *Pieris brassicae* is used as US. In this species, LTM is consolidated after 4 h and there is no ARM in between STM and LTM (Smid et al., 2007; Van den Berg et al., 2011). This form of LTM is transcription-dependent (Smid et al., 2007) and wanes within 5 days after a single conditioning trial (Geervliet et al., 1998); this memory type will be denoted here as Glo-LTM-short. After three spaced conditioning trials, a transcription- and translation-dependent LTM is consolidated within 4 h that lasts at least 5 days, without an ARM (Smid et al., 2007), hence denoted as Glo-LTM-long. The congeneric species *C. rubecula* is a specialist parasitoid of the small cabbage white, *Pieris rapae*. This species forms STM and ARM, which wanes within 24 h after a single oviposition experience. Three spaced conditioning trials are required for transcription-dependent LTM formation, and in this species consolidation is complete after 2–3 days with ARM in between STM and LTM. This memory type, which lasts more than 5 days, will be referred to as Rub-LTM-long. Thus, there is not only a difference in the type of conditioning required for LTM (single or spaced conditioning), but also in the consolidation time (4 h vs. 3 days), in the presence or absence of ARM and in the duration of LTM (short vs. long lasting LTM).

An explanation for this variation may be found in the egg laying behavior of the host species (Smid et al., 2007). The preferred host of the fast learning species *C. glomerata* is *P. brassicae*, and this butterfly lays her eggs in large clusters of up to 150 eggs, on dense stands of host plants of mostly the same species. Finding a caterpillar of this species on a plant reliably predicts many hosts, which may explain the rapid LTM formation of the parasitoid when *P. brassicae* is the US. The host of the slow learning species *C. rubecula* is *P. rapae*, and this butterfly lays single eggs on diverse plant species and flies relatively large distances in between egg laying. Finding a caterpillar of this species on a certain host plant species does not reliably predict that many hosts can be found on that host plant species, and hence *C. rubecula* does not form LTM after one conditioning trial with *P. rapae* as the US. Only after three conditioning trials with *P. rapae* as the US and the same host plant species as CS, LTM is formed. Apparently, the differences in the specific host distribution pattern results in profound different qualities of the two hosts species. This difference would imply that *C. glomerata*, which also accepts *P. rapae* as a host and can successfully develop as larva inside of it, would learn slowly, and form ARM after a single conditioning trial with *P. rapae*, which was indeed shown by Kruidhof et al. (2012). Thus, *C. glomerata* does not form ARM on *P. brassicae*, but after a single conditioning trial on *P. rapae*, *C. glomerata* did form ARM and not LTM. This memory type will be referred to as Glo-ARM.

This natural variation in memory dynamics offers unique possibilities to study inter- and intraspecific variation in gene expression in the brain underlying transcription-dependent memory formation. Our study focuses on the question which genes are involved in the acquisition and consolidation of the different LTM memory types described above. We compared differential expression in 4 memory types: Glo-ARM, Glo-LTM-short, Glo-LTM-long and Rub-LTM-long. For each memory type, we analyzed gene expression levels in the brains without and at different time points after memory induction by means of strand specific, Illumina HiSeq technology. Genes differentially expressed (DE) after conditioning as compared to unconditioned controls could result from memory induction, but may also result from other processes that occur during conditioning, for instance oviposition. Control experiments including CS and US alone and backward pairing are generally used to identify gene expression involved in associative learning. These controls could not be performed here, because the CS and US are in this natural conditioning paradigm very difficult to separate (Bleeker et al., 2006). Instead we made use of the unique possibility offered by the memory type Glo-ARM. Because Glo-ARM formation does not depend on transcription and shares all features in handling during conditioning and subsequent methodology for gene identification with LTM formation, genes that are DE after Glo-ARM induction can be assumed to be unrelated to transcription-dependent LTM formation. We therefore did not consider genes that were DE after LTM induction in the same direction and with the same splice variants as compared to Glo-ARM induction. However, we did consider genes that were DE after LTM induction in opposing direction or with different splice variants as compared to Glo-ARM induction, to reveal genes that are potentially involved in both memory induction and inhibition, or in forgetting. We consider this transcription independent control even more appropriate for the identification of genes involved in LTM formation than US or CS alone or backward pairing; it contains all manipulations and behavior of the treatment groups that form transcription dependent memory, the only difference is that the host species is different.

Currently, there are many genes known to be involved in learning and memory. A recent study on genes induced by transcription dependent learning in the nematode *C. elegans* revealed 757 memory-related genes (Lakhina et al., 2015). Also in our study we hypothesize to find many DE genes related to LTM formation, some of which will be common in each of the LTM memory types, whereas others will be specific to one or two of the LTM memory types. The unique comparison of LTM memory types with ARM may yield genes that are DE expressed in opposing directions, indicating inhibiting mechanisms like forgetting. It should be noted, however, that the approach we use will not reveal genes that are DE in a very small subsets of neurons, since we use brain homogenates for sequencing in which such small amounts of variation cannot be detected. This study is the first to compare inter- and intraspecific conditioning types that result in different memory types in two closely related species. Identifying genes underlying the strong natural variation in memory dynamics in these species will allow studying the evolution of memory formation from an ecological perspective.

## Materials and Methods

### Insect and Plant Rearing

*C. glomerata* and *C. rubecula* laboratory cultures were established from individuals collected in cabbage fields in the vicinity of Wageningen, the Netherlands and reared on *P*. *brassicae* and *P*. *rapae* larvae, respectively. Both *Pieris* species were reared on Brussels sprouts plants (*Brassica oleracea* var. *gemmifera* L. cv. Cyrus). *Nasturtium* plants (*Tropaeolum majus* L. cv. Glorious Gleam) were used to condition the wasps as described below. Insects and plants were reared as described previously (Geervliet et al., 1998; Smid et al., 2007).

To minimize genetic variation all *C. glomerata* samples from a single biological replicate originated from first generation female offspring of one female who mated with one male. All *C. rubecula* samples from a single biological replicate originated from females of a single generation, because one mated female produced too little female offspring in the first generation. All wasps were given unlimited access to water and honey for optimal performance in oviposition learning (Lewis and Takasu, 1990).

### Insect Conditioning Methods

Three leaves of 3–4 week old *Nasturtium* plants were infested with approximately 10 first instar larvae (*P. brassicae* or *P. rapae*) per leaf, 2 days before conditioning of the wasps to induce the production of herbivore-induced plant volatiles. Since oviposition is more easily performed with freshly emerged caterpillars, we replaced these larvae 1 h before conditioning with 15–20 fresh first instar *P. brassicae* larvae, or 10 *P. rapae* larvae per pre-infested leaf, without disrupting the larval feces (Kruidhof et al., 2012). A detailed description of the wasp conditioning procedure has been described previously (Bleeker et al., 2006; Smid et al., 2007). In short, single wasps were brought into contact with the larval feces, close to the larvae, and allowed to oviposit a single larva, where after they were recaptured in a glass tube. Conditioning of a single wasp typically lasted 20 s. Two types of conditioning were applied: (1) a single oviposition trial; or (2) three oviposition trials spaced in time by a 15 min interval. For each trial an unparasitized larva was used, and for spaced conditioning each trial was performed on different infested *Nasturtium* plants. Wasps were kept in a glass tube (75 × 12 mm, VWR) capped with a cotton wool plug in between conditioning trials. After conditioning the wasps were kept in the glass tube until RNA sampling if the RNA was sampled 15 min after conditioning. The wasps were transferred to a rearing cage with water and honey if the RNA was sampled more than 15 min after conditioning. This step was included to prevent that wasps could become hungry or thirsty, because in the oviposition learning paradigm wasps are always provided unlimited access to water and honey.

### Insect Conditioning Types

*C. glomerata* wasps were conditioned with: (1) a single trial on *P. brassicae*, which induces LTM that wanes within 5 days (Glo-LTM-short); (2) three spaced trials on *P. brassicae*, which induces a longer lasting LTM (Glo-LTM-long); and (3) a single trial on *P. rapae*, which induces ARM (Glo-ARM, Table 1). *C. rubecula* was conditioned with three spaced trials on *P. rapae*, which induces ARM and long-lasting LTM (Rub-LTM-long, Table 1). For each of the four conditioning types 20 wasps were conditioned per biological replicate.

**Table 1:** **Description of the conditioning types with the *Cotesia* species, the number of conditioning trials, the host species and the time points after conditioning**.

Conditioning type	*Cotesia* species	# Trials	Host species	Time points
Glo-LTM-short	*C. glomerata*	1	*P. brassicae*	15 m, 1 h, 4 h
Glo-LTM-long	*C. glomerata*	3 spaced	*P. brassicae*	15 m, 1 h, 4 h
Glo-ARM	*C. glomerata*	1	*P. rapae*	15 m, 1 h, 4 h
Rub-LTM-long	*C. rubecula*	3 spaced	*P. rapae*	1 h, 4 h, 24 h
Glo-unconditioned	*C. glomerata*	None	None	None
Rub-unconditioned	*C. rubecula*	None	None	None

*For each time point of conditioning type 3 biological replicates were generated*.

*C. glomerata* wasps were snap frozen in liquid nitrogen 15 min, 1 h and 4 h after conditioning. *C. rubecula* wasps were snap frozen 1 h, 4 h and 24 h after conditioning. Also 20 unconditioned wasps were snap frozen per species and per biological replicate. Three biological replicates were prepared for each time point after conditioning and conditioning type, resulting in 30 samples for *C. glomerata*, i.e., three biological replicates for three conditioning types with three time points each and the unconditioned control, and 12 samples for *C. rubecula*, i.e., three biological replicates for one conditioning type with three time points and the unconditioned control. Note that all handling of wasps was identical between each memory type and time after conditioning, which is important because of the comparison we made between DE genes. Thus the control memory type Glo-ARM was treated exactly the same at all time points as the LTM types.

### RNA Sample Preparation and Sequencing

The heads of the snap frozen wasps were cut with a scalpel and the antennae were removed, where after the heads were transferred to a 1.5 ml microcentrifuge tube, which was stored in liquid nitrogen. All tissues were frozen in the afternoon between 13.30 and 15.00 to avoid circadian variation in expression levels. For each RNA sample 20 heads were collected. We collected intact heads instead of dissected brains to avoid brain damage and RNA degradation, which would occur when dissecting the brain from the head. Dissecting intact brains from *Cotesia* wasps is a relatively slow process due to the tight position of the brain against the head capsule. RNA was extracted from each sample using the RNeasy Micro Kit (Qiagen, Antwerp, Belgium) according to instructions of the manufacturer. RNA quantity and integrity was measured using a 2100 Bioanalyzer (Agilent Technologies, Amstelveen, The Netherlands). One microgram RNA was used for mRNA isolation and subsequent strand-specific mRNA library preparation. One replicate was sequenced by BaseClear BV (Leiden, The Netherlands) using an in-house strand-specific mRNA library preparation protocol, and the other two replicates by the Wageningen Sequencing Facility using the TruSeq Stranded mRNA sample preparation protocol (Illumina). Both sequencing facilities followed the dUTP library preparation method (Parkhomchuk et al., 2009; Levin et al., 2010). Paired-end 100 bp sequencing was performed on a HiSeq2000 platform (Illumina) with TruSeq v3 chemistry. De-multiplexing of obtained sequences was done using CASAVA 1.8.1. software.

### Transcriptome Assembly

The adapters were trimmed from the raw reads using cutadapt (version 0.9.5, options −*O* 10, −*n* 3, −*q* 10) and the reads were quality filtered using fastqmcf (version 1.0, options −*k* 5, −*q* 20, −*l* 50, Table 2). Rather than assembling a transcriptome for each sample, one transcriptome was assembled *de novo* with Trinity (version r2013-02-15, options—SS_lib_type RF (Haas et al., 2013) for each species by pooling the filtered reads of all samples per species (unfiltered transcripts and genes in Table 2). To filter out the transcripts with very little read support, the raw reads of each sample individually were mapped back to the transcriptome using bowtie (version 0.12.7, options −*n* 2, −*e* 99999999, −*l* 25, −3 0, −*a*, −*m* 200, −*I* 1, −*X* 1000,—nofw) and quantified using eXpress (version 1.3.1). The rounded effective read counts per transcript were analyzed with R (version 3.0.8) and only transcripts with more than one read count per million reads for at least three samples were kept in the transcriptome (Table 2). Assembling and filtering the transcriptomes in this way minimizes the risk of missing transcripts with a low read depth in multiple samples.

**Table 2:** **Read numbers before and after quality filtering and adapter trimming, together with statistics of the *de novo* filtered transcriptome assemblies**.

	***C. glomerata***	***C. rubecula***
Raw reads	609.970.373	248.776.799
Filtered reads	596.252.215	239.243.117
Genes	23.287	21.946
With single transcript	17.595	16.768
With multiple transcripts	5.692	5.178
Transcripts	41.182	38.845
Transcriptome size (bp)	30.862.504	29.877.275
N50	3.151	3.425
Maximum transcript length	27.566	27.554

*The N50 and maximum length were determined from the transcripts*.

### Transcriptome Analysis

Transcripts were first aligned using blastx (options: -max_target_seqs 1, -word_size 11, e-value 10) to the annotated proteome of the closest related parasitic wasp *N. vitripennis* (Nvit 2.0). Transcripts that aligned to a protein with less than 60% protein alignment length were aligned using blastx to the NCBI RefSeq nr databases (Sept-01-2013). Transcripts with a protein alignment length of more than 60% to a protein in either the *N. vitripennis* proteome or nr database were defined as protein-coding (sense) transcripts. Transcripts were defined as antisense in two ways: (1) when they aligned with an antisense orientation to a protein in either of the two protein databases with more than 50% protein alignment length; or (2) when they aligned with an antisense orientation to a sense transcript from the same transcriptome as the transcript with more than 80% antisense transcript alignment length and more than 95% sequence identity. The transcripts that were not defined as sense or antisense were aligned to the *N. vitripennis* genome (Nvit 2.0) using blastn (options: -max_target_seqs 1, -word_size 11, e-value 10). Transcripts with more than 80% alignment length and 95% sequence identity were defined as long non-coding RNA (lncRNA). The remaining transcripts were aligned to the NCBI RefSeq nt databases (September, 2013). Again, transcripts with more than 80% alignment length and 95% sequence identity were defined as lncRNA. Transcripts that did not align to a publically available protein or genomic sequence according to the thresholds we defined here embody the unknown fraction of the transcriptome and could be misassembled or (anti)sense transcripts or lncRNA with insufficient homology to known sequences. Putative open reading frames (ORFs) were determined for lncRNA and unknown transcripts using the script “transcripts_to_best_scoring_ORFs.pl” from Trinity (options −m 30 −*S*). Putative ORFs were defined as an ORF with a 5’start and 3’end and minimally 30 amino acids.

### Differential Expression Analysis, Transcriptome Annotation and GO Term Enrichment

DE transcripts were called using the rounded effective counts of each sample, when compared to the unconditioned wasp samples, and a GLM trended dispersion (EdgeR version 3.0.8) with Pearson correlation, taking the replicate effect into account, eight degrees of freedom (12 samples per conditioning type minus four conditioning types) and *P* = 0.05. 3880 *Burkholderia* transcripts that were DE in one replicate in *C. glomerata* after a single trial on *P. rapae* were removed from the transcriptome and expression data to prevent biasing the *C. glomerata* data (Supplementary Figure 1). The multi-dimension scaling plots of the biological coefficients of variation reveal that the gene expression data was affected by a replicate effect (Supplementary Figure 2). We accounted for this by adjusting for any baseline differences between the replicates as described by EdgeR (blocking). The transcriptomes were translated into proteins using the script “transcripts_to_best_scoring_ORFs.pl” from Trinity (options −m 60 −S). The resulting protein fasta sequences were used to do an ortholog search with OrthoMCL (version v2.0.3, 50% alignment length cutoff, E-5 evalue cutoff) using the proteomes of *D. melanogaster* (version 5.53), *A. mellifera* (version 4.5) and *N. vitripennis* (Nvit 2.0). In this way gene names and functions were coupled to the orthologs in our *de novo* assembled *Cotesia* transcriptomes. Following the OrthoMCL analysis, Gene Ontology (GO) terms (version 1.2, 2014-01-10) of *D. melanogaster* (version 2.0) were coupled to the *Cotesia* orthologs. The GO term enrichment analysis was performed with the Blast2go GUI using a Fisher’s exact test, *P* < 0.05. Generic GOSlim categories (GO Consortium, Jan-10-2014) were used to limit the number of GO-term categories.

Following functional annotation of the genes of both species, we limited the DE expression analysis to genes that were DE in any of the three LTM conditioning types that: (1) were not DE in Glo-ARM; or (2) had an opposing expression pattern for all DE transcripts of these genes compared to Glo-ARM, or (3) had other splice variants DE in Glo-LTM-short and Glo-LTM-long than Glo-ARM. We did not analyze splice variation between *C. rubecula* and *C. glomerata*, because splice variant discrimination between species is less accurate due to assembly differences.

## Results

### Transcriptome Assembly

The results of the transcriptome assemblies and filtering of *C. glomerata* and *C. rubecula* are presented in Table 2. Because the number of analyzed samples was higher for *C. glomerata*, the number of reads obtained for *C. glomerata* was almost three times larger than for *C. rubecula*. Nevertheless, both transcriptomes were very similar in terms of size and gene numbers and sizes. Genes with multiple transcripts constituted 24% of the genes identified in the transcriptome and 57% of all transcripts in both *Cotesia* species. The detection of multiple transcripts per gene could result from splice variation, allelic variation, nearly identical paralogs, or misassembled transcripts. We will refer to multiple transcripts per gene as splice variants.

### Structural Annotation of Transcriptomes and DE Transcripts

**Figure 1:**
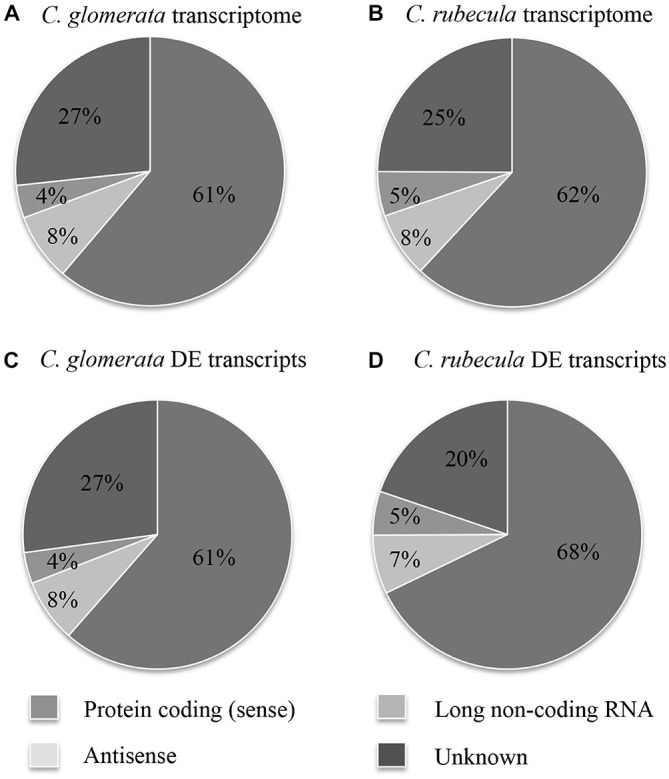
**The fraction of protein-coding (sense) transcripts, antisense transcripts, lncRNA and unknown transcripts is shown for the *C. glomerata* transcriptome (A), the *C. rubecula* transcriptome (B), the DE *C. glomerata* transcripts (C), and the DE *C. rubecula* transcripts (D)**.

The fraction of protein-coding (sense) transcripts, antisense transcripts and lncRNA was determined for both transcriptomes by aligning them to publically available proteome and genome sequences (Figures 1A,B, Supplementary Table 1). These transcriptome fractions were very similar in both species. Strand-specific sequencing revealed that antisense RNA accounted for 8% of the transcriptome. A small portion of the lncRNA and unknown transcripts contained a putative ORF, suggesting these might be (unknown) protein-coding transcripts.

DE transcripts were called for each sample of conditioned wasps by comparison to the transcript expression of unconditioned wasps. The fraction of DE transcripts, divided into sense and antisense transcripts, lncRNA and unknown transcripts are presented in Figures 1C,D and Supplementary Table 2 and were similar to those of the full transcriptomes.

### Functional Annotation of Transcriptomes and DE Transcripts

**Figure 2:**
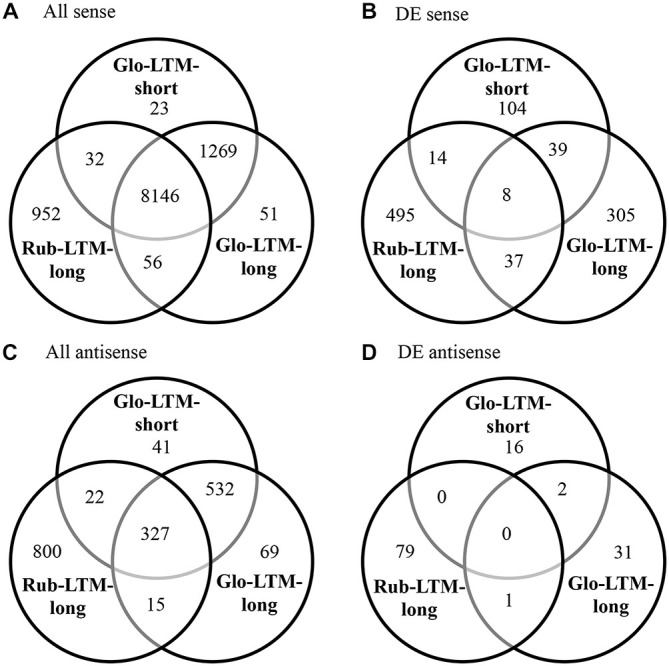
**Venn diagrams of all sense (A), differentially expressed (DE) sense (B), all antisense (C) and DE antisense (D) genes in Glo-LTM-short, Glo-LTM-long and Rub-LTM-long**.

Functional annotation of the genes by OrthoMCL enabled the assessment in overlap between the (DE) transcriptomes of *C. glomerata* and *C. rubecula*. To only assess the overlap in expressed transcripts and not (yet) consider differences in the time after conditioning these transcripts were expressed, we pooled the transcripts of all time points per conditioning type (Figure 2). Although most (78%) of the protein-coding genes were transcribed in both *C. glomerata* and *C. rubecula* (Figure 2A, Supplementary Table 3), only 6% of the DE protein-coding genes were shared between both species (Figure 2B, Supplementary Table 3), suggesting the protein-coding genes related to LTM formation were highly dissimilar. Similarly, the interspecific fraction of genes with DE antisense transcripts was much lower (1%) than that of genes with antisense transcripts in both species (20%, Figures 2C,D, Supplementary Table 3). Comparing the intraspecific gene overlap between both LTM conditioning types of *C. glomerata* revealed that Glo-LTM-long and Glo-LTM-short shared 98% of the protein-coding genes and 85% of the genes with an antisense transcript (Figures 2A,C, Supplementary Table 3). Of the DE protein-coding genes and genes with a DE antisense transcript only 9% and 2% were shared between Glo-LTM-short and Glo-LTM-long, respectively (Figures 2B,D, Supplementary Table 3).

### Antisense Transcription

**Figure 3:**
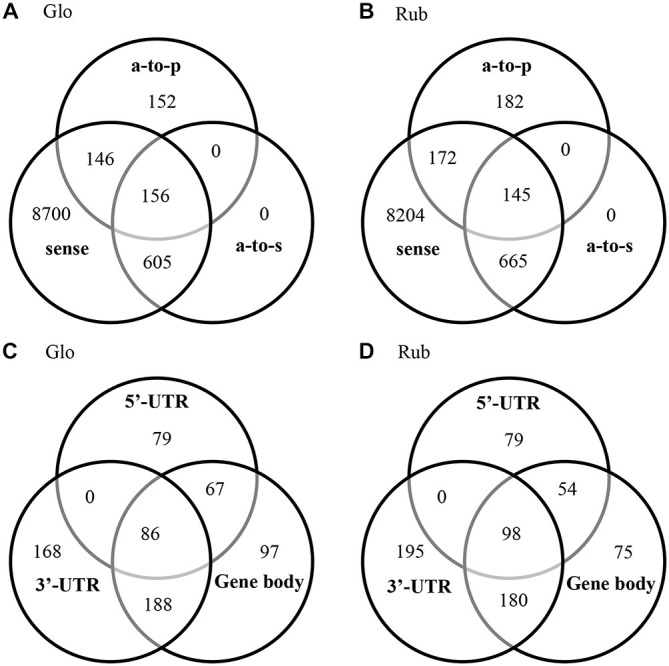
**Venn diagrams of the genes with a sense transcript, antisense-to-protein (a-to-p) transcript and antisense-to-sense (a-to-s) transcript in *C. glomerata* (Glo, A) and *C. rubecula* (Rub, B)**. Venn diagrams of alignment of the antisense-to-sense transcripts to (part of) the 5′-UTR region, (part of) the protein-coding region (coding) and (part of) the 3′-UTR region in *C. glomerata* (Glo, **C**) and *C. rubecula* (Rub, **D**) of sense transcripts that align to *N. vitripennis* proteins with more than 90% protein coverage. Only the antisense-to-sense transcripts that align to the 5’-UTR, protein-coding region and 3’-UTR, align to the full protein-coding region.

Antisense transcripts were observed for 9.4 and 11% of the annotated genes in *C. glomerata* and *C. rubecula*, respectively (Figures 3A,B, Supplementary Table 4). We discriminated three types of antisense transcripts: (1) transcripts with a reverse orientation to a protein (antisense-to-protein transcripts); (2) transcripts with a reverse orientation to a sense transcript (antisense-to-sense transcripts); and (3) transcripts with a reverse orientation to both a protein and sense transcript (Supplementary Tables 1, 2). To assess the occurrence of these types of antisense transcripts in the brain transcriptomes of both species, we pooled the antisense transcripts of all time points and all conditioning types per species (Figures 3A,B). The fraction of antisense transcripts that was categorized as both antisense-to-protein and antisense-to-sense was only 11–14% (Figures 3A,B, Supplementary Table 4). This has two reasons. First, a large fraction of genes with antisense-to-protein transcripts had no sense transcripts (Figures 3A,B, Supplementary Table 4), suggesting a gene-transcription-inhibiting mechanism by antisense-to-protein transcripts. Second, the majority of the antisense-to-sense transcripts did not align to the (majority of the) protein-coding region of sense transcripts (Figures 3C,D, Supplementary Table 5). Most of the antisense-to-sense transcripts (52–55%) aligned to the 3′-UTR with or without part of the protein-coding region, whereas 20–21% of the antisense-to-sense transcripts aligned to the 5′-UTR with or without part of the protein-coding region.

**Figure 4:**
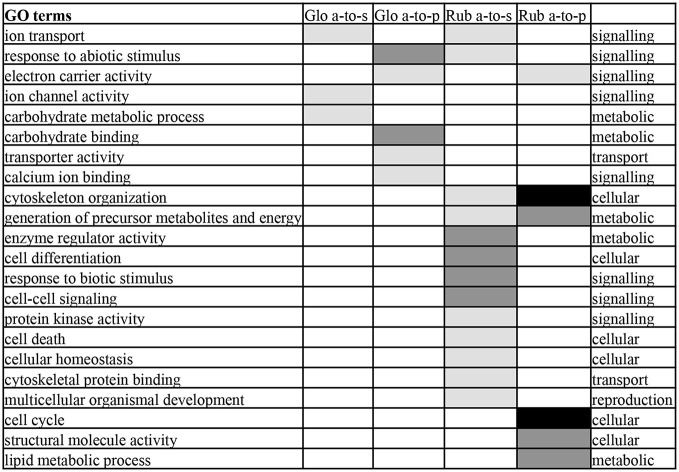
**Enriched Gene Ontology (GO) terms of the categories biological process and molecular function in antisense-to-protein (a-to-p) and antisense-to-sense (a-to-s) transcripts in** *C. glomerata* **(Glo) and** *C. rubecula* **(Rub)**. GO terms enriched with *P* < 0.001 are indicated in black, with 0.01 < *P* < 0.001 in dark gray, 0.05 < *P* < 0.01 in light gray.

To determine what gene functions were affected by antisense transcription, GO term enrichment analysis of the genes with antisense-to-sense and antisense-to-protein transcripts was performed (Figure 4). Because of the large overlap in antisense transcripts between Glo-LTM-short and Glo-LTM-long (Figure 2C), GO term enrichment analysis of antisense transcription was performed per species, rather than per conditioning type. Three GO terms emphasized on signaling in both *C. glomerata* and *C. rubecula*, i.e., ion transport, electron carrier activity and response to abiotic stimulus (Figure 4). Carbohydrate metabolism-related GO terms were more specific to *C. glomerata*, whereas the other metabolic related GO terms and all cellular and reproduction related GO terms were specifically enriched in *C. rubecula*. Because signaling-related GO terms are of interest in our study on memory formation and because of the apparent overlap of a number of signaling related GO terms between both *Cotesia* species, we considered all antisense transcripts underlying the signaling-related enriched GO terms (Supplementary Table 6). These were 176 antisense transcripts of which 124 were present in either *C. glomerata* (33) or *C. rubecula* (91). Among the proteins that underlie these signaling-related antisense transcripts were membrane proteins (23%), like receptors, channels, antiporters and vacuole ATPases. Two other well-represented types of proteins with antisense transcripts were kinases (10%, 18 out of 176) and proteins involved in calcium dependent signaling (10%, 17 out of 176).

### Time-Dependent Differential Expression Analysis

**Figure 5:**
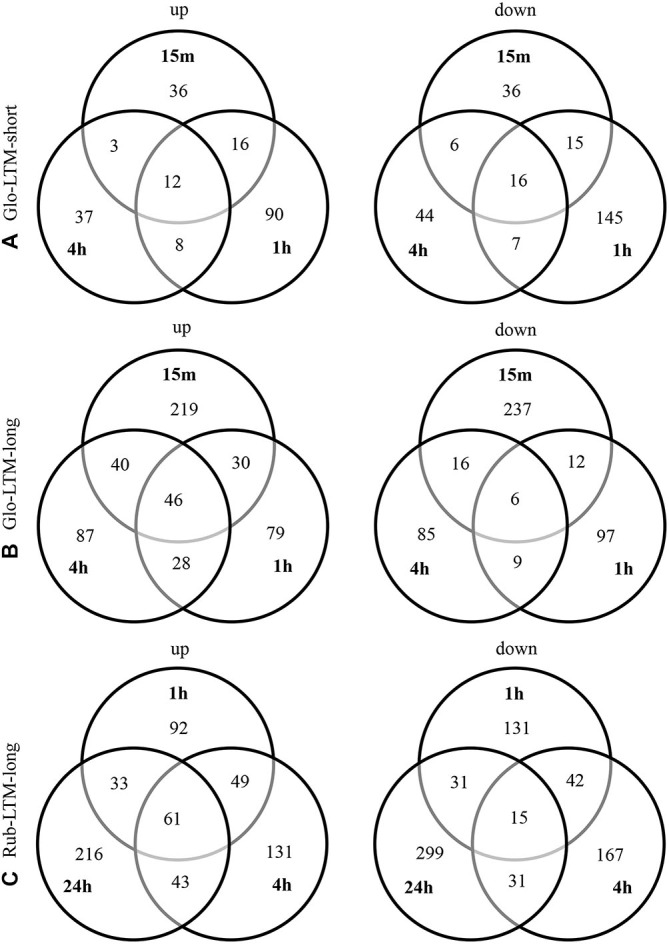
**Venn diagrams of the up- and downregulated transcripts at each indicated time point in Glo-LTM-short (A), Glo-LTM-long (B) and Rub-LTM-long (C)**.

The highest numbers of DE transcripts were observed 1 h after Glo-LTM-short conditioning (66%), 15 min after Glo-LTM-long conditioning (61%), and 24 h after Rub-LTM-long conditioning (54%) (Figure 5, Supplementary Tables 7, 8). This difference may reflect the fast (within 4 h) LTM consolidation in *C. glomerata* and slow (2–3 days) LTM consolidation in *C. rubecula*. Given the fact that the conditioning procedure of Glo-LTM-long lasts approximately 30 min longer than that of Glo-LTM-short, both single and spaced LTM in *C. glomerata* were likely to have most transcripts DE at the same time after LTM initiation, i.e., approximately 1 h after the first trial.

Most DE transcripts were not shared between time points in a single conditioning type, whether they were sense or antisense transcripts or lncRNA, indicating that gene expression is different at the measured time points after conditioning and suggesting all transcript categories had a quick turn-over rate.

### Differentially Expressed Sense Transcripts

We only consider DE genes that were related to LTM formation by retaining genes that were DE in any of the three LTM conditioning types that: (1) were not DE in Glo-ARM; or (2) had an opposing expression pattern for all DE transcripts of these genes compared to Glo-ARM; or (3) had other splice variants DE in Glo-LTM-short and Glo-LTM-long than Glo-ARM. These genes can potentially discriminate between LTM and ARM formation.

Alternative splicing was abundant among the DE sense transcripts. Whereas only 24% the genes in the *Cotesia* transcriptomes had more than one transcript, most of the DE genes had multiple transcripts, i.e., 71% in *C. glomerata* and 67% in *C. rubecula* (Supplementary Table 9). Fifteen percent of the DE genes had multiple DE transcripts (Supplementary Table 9).

#### Comparison of DE Genes with Different Splice Variants or with Opposing Expression Between LTM and ARM

Of the 232 DE genes with multiple DE splice variants in *C. glomerata*, 97 genes had one or multiple different splice variants DE between LTM and ARM, 52 genes between Glo-LTM-short and Glo-LTM-long and 17 genes between all three *C. glomerata* conditioning types (Supplementary Table 10). Examples of genes with different splice variants DE between LTM and ARM are *Rho1*, a Ras related GTPase, and Pelle (*ple*), a protein kinase involved in the Toll signaling pathway, which both had one splice variant downregulated in Glo-LTM-short and Glo-LTM-long and another splice variant downregulated in Glo-ARM. Rad, Gem/Kir family member 1 (*Rgk1*), a GTPase signaling protein, is an example of a gene with different splice variants DE in all three conditioning types. Darkener of apricot (*doa*), a protein kinase involved in alternative splicing and microtubule transport, had two upregulated and one downregulated transcript in Glo-LTM-long, whereas one of these upregulated transcripts was downregulated in Glo-ARM.

Only 28 genes had an opposing expression pattern of all DE transcripts between LTM and ARM, of which five between Glo-LTM-short and Glo-ARM, 15 between Glo-LTM-long and Glo-ARM and 8 between Rub-LTM-long and Glo-ARM (Supplementary Table 11). Most (17) of the 20 genes with an opposing expression pattern between LTM and ARM in *C. glomerata* had different splice variants DE between LTM and ARM. Two examples of such genes are Protein kinase C δ (*PKCδ*), which was upregulated in Glo-LTM-long and downregulated in Glo-ARM, and Histone demethylase 4B (*Kdm4B*), which was downregulated in Glo-LTM-long and upregulated in Glo-ARM. These results suggest a role for alternative splicing in differentiating between memory types and in particular between LTM and ARM.

#### Comparison of DE Genes Between Different Types of LTM Formation

**Figure 6:**
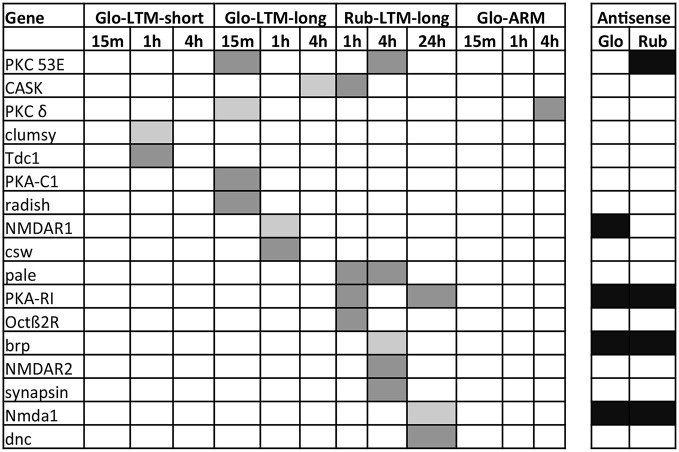
**Expression of the DE protein-coding genes involved in memory formation from literature at each time point after conditioning in all four *Cotesia* conditioning types**. Upregulated transcripts are indicated in light gray, downregulated in dark gray. Genes with antisense transcripts are indicated in black for *C. glomerata* (Glo) and *C. rubecula* (Rub).

**Figure 7:**
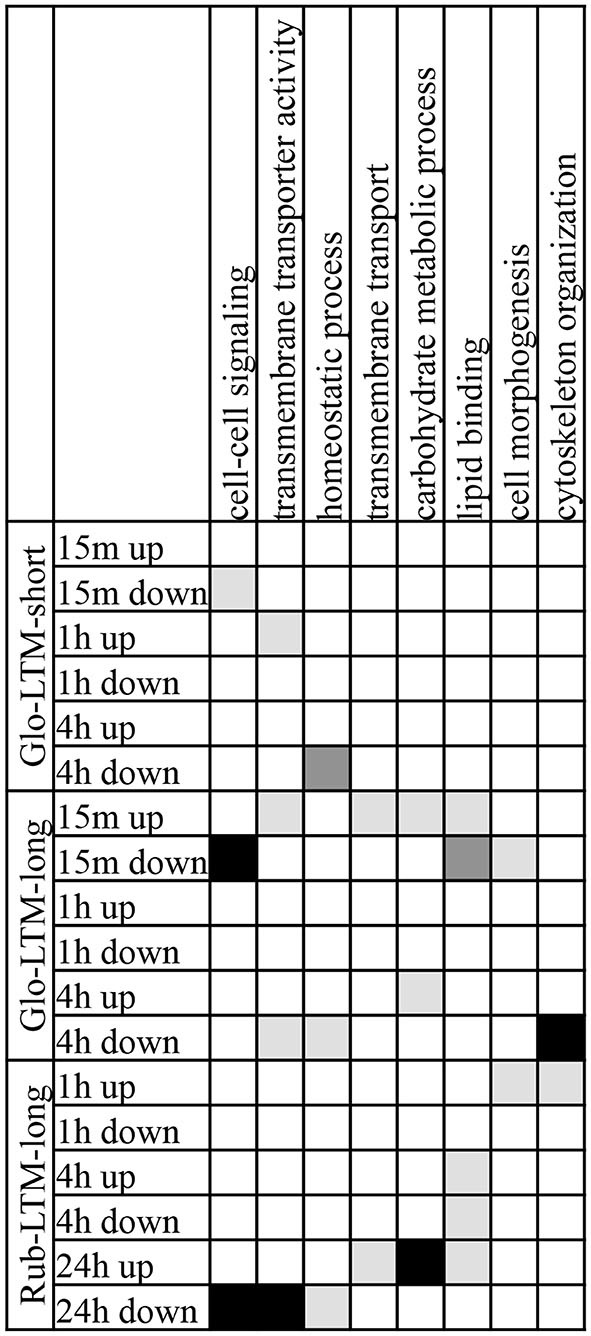
**GO terms, each with 10 or more genes, of the categories biological process and molecular function that were enriched in two or three LTM conditioning types in up- and downregulated transcripts at each time point after conditioning**. GO terms enriched with *P* < 0.001 are indicated in black, with 0.01 < *P* < 0.001 in dark gray, 0.05 < *P* < 0.01 in light gray.

To identify candidate genes responsible for variation in LTM formation, we compared DE transcripts between Glo-LTM-short and Glo-LTM-long (intraspecific variation) and Glo-LTM-long and Rub-LTM-long (interspecific variation) that were not DE in Glo-ARM. We analyzed these DE protein-coding transcripts using three strategies. First, we identified genes with a similar expression pattern in terms of up- or downregulation in Glo-LTM-short and Glo-LTM-long and genes with a similar expression pattern in Glo-LTM-long and Rub-LTM-long (Supplementary Table 12). As a second, complementary approach we assessed the DE expression of 79 genes with a known function in memory formation (Supplementary Table 13). Seventeen of these genes were DE during LTM formation in *Cotesia*, of which three were DE in multiple conditioning types (Figure 6). Third, we performed a GO term enrichment analysis of the DE genes at each time point and for each LTM conditioning type (Supplementary Table 14, Figure 7). This latter analysis was used to describe both the shared and unique GO terms and genes between the LTM conditioning types.

##### Intraspecific variation in LTM formation

Intraspecific variation in gene expression between Glo-LTM-short and Glo-LTM-long originates from a single vs. three spaced oviposition trials of *C. glomerata* on *P. brassicae* larvae, respectively. As a result Glo-LTM-short wanes within 5 days, whereas Glo-LTM-long lasts more than 5 days.

Only 47 genes were DE in both Glo-LTM-short and Glo-LTM-long (Figure 2B). Nearly all (45) of these shared genes had a similar expression pattern in both conditioning types and are therefore potential LTM inducing genes in *C. glomerata* (Supplementary Table 12). Five shared genes were involved in Ras related GTPase signaling and were all downregulated, i.e., *Rho1*, Rab escort protein (*Rep*), Still life (*sif*), Ran binding protein 16 (*Ranbp16*) and SET domain binding factor (*Sbf*). Another gene involved in Ras related GTPase signaling with a role in memory formation was Radish (*rad*), which was only DE in Glo-LTM-long (Figure 6). Eight of the 47 shared genes had different splice variants expressed in both conditioning types, of which *olf186-F*, a component of a calcium channel, is an example (Supplementary Table 10).

Analysis of known memory genes revealed five genes involved in memory formation that were only DE in Glo-LTM-long (Figure 6). Besides *rad*, we identified corkscrew (*csw*), which is a protein tyrosine phosphatase, NMDA receptor 1 (*NMDAR1*), *PKCδ*, and a catalytic subunit of Protein kinase A (*PKA-C1*). Two genes were only DE in Glo-LTM-short. These are *clumsy*, which is a glutamate-gated ion channel, and Tyrosine decarboxylase 1 (*Tdc1*), an enzyme involved in dopamine (DA) synthesis (Figure 6).

The GO term enrichment analysis revealed that three GO terms were enriched in Glo-LTM-short and Glo-LTM-long, i.e., cell-cell signaling, homeostatic process and transmembrane transporter activity (Figure 7). Cell-cell signaling was enriched 15 min after Glo-LTM-short and Glo-LTM-long conditioning, which reflects the immediate response of cell-cell signaling related genes to LTM conditioning in *C. glomerata*. Homeostatic process, on the other hand, was enriched 4 h after Glo-LTM-short and Glo-LTM-long conditioning, which implies the late response of genes involved in maintaining an internal steady state. Transmembrane transporter activity, an aspect of intracellular signaling, revealed an intermediate response in Glo-LTM-short (1 h) and Glo-LTM-long (15 min and 4 h). Of the 36 genes underlying these three GO terms, 7 genes were DE in both conditioning types (Supplementary Table 15). *Rep*, *sif* and *olf186-F* are examples of shared genes underlying these shared GO terms. The remaining 29 genes were uniquely DE in either Glo-LTM-short or Glo-LTM-long; examples that are known to be involved in memory formation are *Clumsy*, *PKCδ* and *PKA-C1* (Supplementary Table 15, Figure 6).

##### Interspecific variation in LTM formation

Interspecific variation in gene expression between Glo-LTM-long and Rub-LTM-long originates from three spaced oviposition trials of *C. glomerata* on *P. brassicae* larvae vs. *C. rubecula* on *P. rapae* larvae, respectively. Glo-LTM-long is consolidated within 4 h, whereas Rub-LTM-long is consolidated after 2–3 days.

Only 45 genes were DE in both Glo-LTM-long and Rub-LTM-long out of the 389 DE genes in Glo-LTM-long and 554 DE genes in Rub-LTM-long (Figure 2B). Thirty-five of these genes had a similar expression pattern between Glo-LTM-long and Rub-LTM-long (Supplementary Table 12). These genes are potentially involved in LTM formation in both species.

A number of genes involved in memory formation were DE in both conditioning types (Figure 6). Protein C kinase 53E (*PKC 53E*) and *PKA* were both downregulated in both conditioning types, though of the latter gene a catalytic subunit was downregulated in Glo-LTM-long (*PKA-C1*) and a regulatory subunit in Rub-LTM-long (*PKA-R1*). Another example of a gene with different subunits DE in both conditioning types is the NMDA receptor, which has subunit 1 (*NMDAR1*) upregulated in Glo-LTM-long and subunit 2 (*NMDAR2*) downregulated in Rub-LTM-long. Another gene with an opposing expression pattern was Ca^2+^/calmodulin-dependent serine protein kinase (*CASK*). Genes that are involved in memory formation, but were only DE in one conditioning type, were *PKCδ*, *rad* and *csw*, which were only DE in Glo-LTM-long, and cAMP phosphodiesterase Dunce (*dunce*), tyrosine hydroxylase (TH) Pale, Octopamine β2 receptor (*Octβ2R*), Bruchpilot (*brp*) and Synapsin, which were only DE in Rub-LTM-long (Figure 6). This interspecific variation in gene expression may be involved in the observed differences in LTM consolidation.

The GO term enrichment analysis revealed 8 GO terms that were enriched in both Glo-LTM-long and Rub-LTM-long (Figure 7). The majority of the GO terms were enriched in Glo-LTM-long with an immediate response (15 min) after conditioning, whereas in Rub-LTM-long they had a late response (24 h) after conditioning. Exceptions were transmembrane transporter activity and carbohydrate metabolic process that also had a late response in Glo-LTM-long, and homeostatic process and cytoskeleton organization that only had a late response in Glo-LTM-long. In addition, cytoskeleton organization and cell morphogenesis had an early response in Rub-LTM-long. Only 11 of the 94 genes underlying these 8 GO terms were DE in both conditioning types, for example *PKC 53E* (Supplementary Table 15).

Although 19 GO terms were unique to either Glo-LTM-long or Rub-LTM-long (Supplementary Table 14), they indicate an enrichment of similar cellular processes, for example cell communication and cell-cell signaling or lipid metabolic process and lipid binding. Furthermore, many of these GO terms were related to neuronal signaling and morphogenesis, for example neurological system process or cytoskeletal protein binding. We listed the DE genes underlying the most significantly enriched GO terms in Glo-LTM-long and Rub-LTM-long in Supplementary Table 16. Neurological system process, the most significantly enriched GO term in Glo-LTM-long, had 12 out of the 19 genes only DE in Glo-LTM-long, among which *PKA-C1*, *PKC53E*, *CASK* and *rad*. Cytoskeletal protein binding, the most significantly enriched GO term in Rub-LTM-long, had 14 out of the 18 genes only DE in Rub-LTM-long.

### Differentially Expressed Antisense Transcripts

Only 3–4% of the antisense transcripts were up- or downregulated in *Cotesia* (Supplementary Tables 1, 2). Unfortunately, the function and/or name of many genes that these DE antisense transcripts aligned to were unknown (Supplementary Table 17). A gene important for neuronal signaling is the *WNK* gene, which had a downregulated antisense transcript in Glo-LTM-long. A small GTPase of the Rab family, *Rab8*, which is thought to be involved in neuronal protein transport, had an upregulated antisense transcript in Glo-LTM-long. An antisense transcript of Calmodulin (*Cam*), a gene involved in LTM formation, was downregulated 4 h after Rub-LTM-long induction. Though antisense transcripts formed a substantial part (7–8%) of the transcriptomes and DE transcripts following LTM induction (Figure 1), and a number of DE antisense transcripts are involved in neuronal signaling, the exact role of antisense transcription in LTM formation remains to be determined.

## Discussion

In this study, we compared learning induced gene transcription in the brains of two parasitic wasp species that express three types of LTM that differ in consolidation time and duration, i.e., Glo-LTM-short (consolidated in 4 h, lasts < 5 days), Glo-LTM-long (consolidated in 4 h, lasts > 5 days) and Rub-LTM-long (consolidated in 2–3 days, lasts > 5 days). This comparison is unique because it links specific variation in gene expression to ecologically relevant variation in memory types within and between the wasp species and between the uses of different host species as US. We aim to describe these genes below according to their known functions in memory formation in other organisms, especially *D. melanogaster* and *A. mellifera*, but also in mammals. Thereafter we discuss our main conclusions in the context of natural variation in learning and memory.

### Protein Kinases and Phosphatases

The importance of several protein kinases and phosphatases in learning and memory formation is well documented in vertebrates (Giese and Mizuno, 2013), *D. melanogaster* (Margulies et al., 2005) and *A*. *mellifera* (Eisenhardt, 2014). The cAMP-dependent protein kinase A (*PKA*) plays a central role in all model species, and prolonged *PKA* activity is required for LTM. This central role for *PKA* in learning and memory formation is reflected in *Cotesia* as well, since we found DE transcripts of *PKA* in all memory types except Glo-LTM-short.

Protein kinase C (*PKC*) is another kinase important to learning and memory (Grünbaum and Müller, 1998). This family of kinases consists of three subclasses, i.e., classical (*cPKC*), novel (*nPKC*) and atypical (*aPKC*), according to the requirements for activation. In insects, *aPKCζ* has most often been linked to memory formation and memory maintenance (Deng et al., 2014). Recently, *PKC98E*, the *Drosophila* *nPKCδ* homologue, was shown to be involved in the *Notch* signaling pathway to induce hyperphosphorylation of transcription factor *CREB*, thereby regulating LTM formation (Zhang et al., 2013). In *Cotesia* two *PKC*s were DE, i.e., *cPKC53E* and *nPKCδ*. Interestingly, the latter gene had two transcripts DE of which one was downregulated in Glo-ARM and upregulated in Glo-LTM-long and another only upregulated in Glo-LTM-long. Recently, we also observed variation in *PKC* expression discriminating between LTM and ARM in the parasitic wasp genus *Nasonia* (Hoedjes et al., 2015). After a single host encounter *N. vitripennis* forms LTM, whereas *N. giraulti* forms ARM (Hoedjes and Smid, 2014). Only in *N. vitripennis* an *aPKC* was upregulated, whereas this gene was not DE in *N. giraulti*.

A third kinase that plays a key role in memory formation is Ca2+/calmodulin kinase II (*CaMKII*), a serine/threonine kinase that is activated by autophosphorylation upon Ca2+/calmodulin signaling (Malik and Hodge, 2014). This process is inhibited by a Ca2+/*CASK*, which acts on inhibitory phosphorylation sites of *CaMKII*. Both *CASK* and *CaMKII* are required for ARM and LTM aversive memory in *Drosophila*. Whereas we did not observe DE of *CaMKII* in *Cotesia*, there was a remarkable contrast found in DE of *CASK*, which was upregulated in Glo-LTM-long but downregulated in Rub-LTM-long.

A number of phosphatases involved in memory formation were DE in *Cotesia* after conditioning. SHP2 phosphatase (*corkscrew*), which was downregulated in Glo-LTM-long, affects the length of the inter-trial interval between multiple conditioning trials that is required to form LTM in *Drosophila*. Upregulation of this gene in the mushroom bodies was shown to shorten this interval, thus resulting in LTM after massed conditioning trials (Pagani et al., 2009).

The cAMP phosphodiesterase *dunce* was downregulated in Rub-LTM-long. In *Drosophila* this was one of the first memory genes identified by mutant screens (Tully and Quinn, 1985). PDE6 is a phosphodiesterase involved in regulation of intracellular levels of cGMP (Day et al., 2005). The cGMP signaling pathway is involved in natural variation in LTM formation in *Drosophila*, as shown by studies on the cGMP-dependent protein kinase *foraging* (Mery et al., 2007). This gene was downregulated in Glo-LTM-short and Glo-LTM-long.

Using a courtship rejection memory assay, (Winbush et al., 2012) compared naive and LTM memory types in *D. melanogaster* using short-read deep-sequencing. They found many kinases and phosphatases involved in memory formation that were also revealed by our study, such as *dunce*, *PKA-R1* and *PKC*.

### Glutamate Receptor and Calcium Channels

In vertebrates, the activity of the glutamate-gated Ca2+-channel NMDA receptor is thought to underlie coincidence detection of neuronal activity, and is thereby regarded as the key to Hebbian learning involving synaptic plasticity (Tabone and Ramaswami, 2012). In *D. melanogaster*, this channel, which consists of subunits Nmdar1 and Nmdar2, was shown to be specifically involved in LTM (Wu et al., 2005), by regulating the expression of an inhibitor isoform of *CREB*, *dCREB2b* (Miyashita et al., 2012). In honeybees, the NMDA receptor homologue *AmNR1* subunit was shown to be involved in eLTM (Müssig et al., 2010; Leboulle, 2013). We also observed DE of these subunits, suggesting that they play a role in LTM formation in *Cotesia* as well, since *Nmdar1* was upregulated in Glo-LTM-long, whereas *Nmdar2* was downregulated in Rub-LTM-long.

*Olf186-F* (dOrai), a structural component of a Ca2+ release-activated Ca2+ channel (*CRAC*), is involved in regulation of Ca2+ release from the endoplasmic reticulum (Lewis, 2011). In mice, these channels have been implicated in the stabilization of dendritic spines, which are structures thought to be important in memory, by generating sustained Ca^2+^ signals required for activating *CAMKII* (Sun et al., 2014). In *Cotesia* the importance of *olf186-F* for LTM is suggested because it was upregulated in Glo-LTM-short and Glo-LTM-long, whereas another splice variant was downregulated in Glo-LTM-long.

### Small GTPases and Related Genes

Synaptic plasticity underlies memory formation, and changes in synaptic properties are required to store memory, but also to forget (Berry and Davis, 2014). The cytoskeleton, constituted by actin microfilaments and microtubules, is one of the critical components of synaptic plasticity. We found many genes DE in *Cotesia* that are related to mechanisms that remodel the cytoskeleton and are involved in synaptic plasticity. In particular, we found many DE genes among members of the superfamily of small RAS GTPases, which have diverse roles as molecular switches in numerous cellular processes (Correll et al., 2008). These GTPases are active when bound to GTP and inactive when bound to GDP. Guanine nucleotide exchange factors (GEFs) control the activity of GTPases. Interestingly, we observed many cases of different splice variant expression and opposing expression patterns between Glo-ARM and any of the LTM memory types in both small GTPases and GEFs in *Cotesia*. For instance, Rho GTPases are key regulators involved in the stability of dendritic branches by regulating the actin and microtubule cytoskeleton (Van Aelst and Cline, 2004). *Rho1* (*RhoA*) restricts neurite outgrowth in mushroom body neurons in *D. melanogaster* (Lee et al., 2000). The activity of Rho GTPases is regulated by GEFs like *trio* (Iyer et al., 2012). In *Cotesia* one transcript of *Rho1* was downregulated in Glo-LTM-short and Glo-LTM-long, whereas another splice variant was downregulated in Glo-ARM. Similarly, one splice variant of *trio* was downregulated in Glo-LTM-short and Glo-ARM, whereas another splice variant was downregulated in Glo-LTM-long. Another GEF gene, *still life (sif)*, a *Drosophila* homologue of Tiam (Tolias et al., 2011), was downregulated in Glo-LTM-short and in Glo-LTM-long. An example of GTPases with opposing DE between ARM and LTM is *Rab40*. This is a GTPase involved in regulation of membrane trafficking, and thereby in regulation of transport of vesicles towards the synapse (Hutagalung and Novick, 2011). This GTPase was upregulated in Glo-LTM-short and downregulated in Glo-ARM. Rap GTPase activating protein *Radish* is specifically required for ARM formation in odor-shock learning in *Drosophila* (Folkers et al., 2006). It was downregulated immediately after conditioning in Glo-LTM-long, which may indicate a role in the suppression of ARM in *C. glomerata*. All these examples suggest both gene-specific and splice variant-specific differentiation of GTPases and GEFs between LTM and ARM memories. A similar result was observed in *Nasonia*, where many RAS related signaling genes were DE during LTM formation in *N. vitripennis*, but not during ARM in *N. giraulti* (Hoedjes et al., 2015).

### Splicing and Transport of mRNA

Within a neuron, mRNA can be spliced into different transcripts, which can have different or even opposite functions (Lee et al., 2003). Furthermore, mRNA can be transported to synapses in an asymmetrical manner, to allow synapse-specific local protein synthesis. These two mechanisms of mRNA splice variation and localization have a crucial role in experience-dependent synaptic plasticity, and hence in learning and memory formation (Hutten et al., 2014). Tropomyosin I and II are two tightly linked genes which regulate actin filament function, and have many different splice forms (Gunning, 2008). In *Cotesia* splice variants of Tropomyosin I were upregulated in Glo-LTM-long and Glo-ARM and a different splice variant was downregulated in Glo-ARM. Tropomyosin II has a role in the *Staufen* pathway of mRNA translocation (Gardiol and St Johnston, 2014). *Staufen* is an RNA binding protein, and the *Staufen* pathway is required for LTM in *Drosophila* (Dubnau et al., 2003). *Moesin* is an actin binding protein required for proper localization of *Staufen* (Dubnau et al., 2003). In *Cotesia* we found three different splice variants of *Moesin* DE in Glo-LTM-long and Rub-LTM-long.

Five other genes with a role in splice variation were DE in *Cotesia*. *Darkener of apricot* (*doa*), a protein kinase involved in regulation of alternative splicing (Kpebe and Rabinow, 2008), was upregulated in Glo-LTM-long and had the same splice variant downregulated in Glo-ARM. *Armadillo*, a transcriptional regulator in the canonical Wnt pathway, required for LTM in *Drosophila* (Tan et al., 2013), was downregulated in Glo-LTM-short. Three genes involved in splice variation were DE in Glo-LTM-long, i.e., *Ataxin-*2 b*inding protein 1* (*A2bp1*), which is involved in splicing of exons in ion channels, receptors and synaptic proteins (Lee et al., 2009), *Mushroom-body expressed* (*Mub*) (Park et al., 2004), which is involved in mRNA splicing, and *Parg* (Poly(ADP-ribose) glycohydrolase), which modulates alternative splicing (Ji and Tulin, 2009).

### Epigenetic Regulation

Chromatin remodeling, which concerns modification of histones and DNA, is involved in LTM formation, because it can semi-permanently change the expression levels of genes responsible for memory consolidation bidirectionally (Zovkic et al., 2013). We found several genes involved in these processes DE in *Cotesia*.

Histone methylation regulates memory formation in mammals and flies (Gupta et al., 2010). In *Cotesia* we found that *Histone demethylase 4B* was downregulated immediately after Glo-LTM-long induction, whereas another splice variant was upregulated in Glo-ARM after 4 h. Another histone demethylase is *Absent, small, or homeotic discs 1* (*Ash1*), which was also downregulated in Glo-LTM-long, but only after 4 h. *Ash1* is associated with *kismet*, an ATP-dependent chromatin remodeler, which is required for memory formation (Melicharek et al., 2010) and DE in Glo-LTM-short and Glo-LTM-long. *Rm62* is an RNA helicase involved in alternative splicing and transcriptional activation, by recruiting the histone methyl transferase *SU(VAR)3–9* (Boeke et al., 2011). In *Cotesia* we found two splice variants of *Rm62* DE in Glo-LTM-long and Rub-LTM-long.

Histone acetylation is another important regulatory mechanism involved in memory related gene expression (Merschbaecher et al., 2012; Gräff and Tsai, 2013). *Enhancer of yellow 2* (*e(y)2*) is a transcriptional activator and member of the histone acetyltransferase *SAGA* complex (Kopytova et al., 2010). One splice variant of this gene was downregulated in Glo-LTM-short whereas another splice variant was downregulated in Glo-LTM-long. Two histone deacetylases were upregulated in Rub-LTM-long, i.e., *HDAC4* and *HDAC6*. In *Drosophila*, the role of *HDAC4* in LTM has been demonstrated recently (Fitzsimons et al., 2013). *Molybdenum cofactor synthesis 2* (*Mocs2*), which was upregulated in Glo-LTM-long, is part of the *ATAC* histone acetylase complex and is related to MAPK signaling (Suganuma et al., 2010).

Besides histone modifications, also repositioning of histones and modification of DNA itself can regulate transcriptional activity. DNA methylation is a key regulator of LTM (Day and Sweatt, 2010). *Methyltransferase 2* (*Mt2*) is a DNA methyltransferase that was downregulated in Glo-LTM-long. The chromatin remodeler, *ATP-dependent chromatin assembly factor 1* (*Acf1*) is an essential element of the *ISWI* ATP-dependent chromatin remodeling complex and involved in the control of the *Wingless/Wnt* signaling pathway (Liu et al., 2008). This gene had a downregulated antisense transcript in Glo-LTM-long.* Mep-1* is a component of another chromatin remodeling complex, the *NuRD* complex, which has both chromatin remodeling and HDAC activity (Reddy et al., 2010). In *Cotesia* *Mep-1* was upregulated in Glo-LTM-long and Rub-LTM long.

### Neurotransmitter and Neuropeptide Synthesis and Receptors

The importance of OA and DA in memory formation is well documented in insects. OA is a monoaminergic messenger that mediates the reward in appetitive conditioning in *A. mellifera (Hammer, 1997)*, the cricket *Gryllus bimaculatus* (Unoki et al., 2006) and in *D. melanogaster* (Schwaerzel et al., 2003). Two classes of receptors are known, the α-type, which activates intracellular Ca2+ release, and the β-type, which activates cAMP (Balfanz et al., 2013). The α-type receptors have mostly been linked to appetitive memory in honeybees (Farooqui et al., 2003) and *D. melanogaster* (Kim et al., 2013), but recently also a role for *Oct2β* was demonstrated in modulation of appetitive learning in *D. melanogaster* (Burke et al., 2012). In *Cotesia* we found that the *Oct2β* receptor was downregulated in Glo-LTM-long and Rub-LTM-long, whereas the *Oct1β* receptor was downregulated in Glo-ARM. A role for the latter receptor in learning has so far not been demonstrated. Tyrosine decarboxylase, an enzyme required for OA synthesis was downregulated in Glo-LTM-short. The prominent role of DA signaling in mediating the reward in appetitive learning has more recently been discovered in *D. melanogaster* (Kim et al., 2007; Waddell, 2013), whereas in *A. mellifera* DA seems to play an inhibiting role in appetitive learning (Klappenbach et al., 2013). In *Cotesia* TH, an enzyme involved in DA synthesis was downregulated in Rub-LTM-long.

*Adipokinetic hormone* (*AKH*) has a role in mediation of motivation for learning in *Drosophila* (Gruber et al., 2013). This neuropeptide was shown to modulate the odor-conditioned response using food as a reward, but not using electroshock as punishment. In *Cotesia* we found that the *AKH* receptor was upregulated in Rub-LTM-long and downregulated in Glo-ARM.

*Neprilysin 3* (*Nep3*) is a synaptic peptidase involved in inactivation of neuropeptides (Isaac et al., 2007). Interestingly, it also degrades the Aβ peptide involved in Alzheimers disease. Overexpression of human NEP in a *Drosophila* model of Alzheimer’s disease reduced Aβ peptide deposits, but also inhibited *CREB* mediated transcription (Iijima-Ando et al., 2008). In *Cotesia* *Nep3* was downregulated in Glo-LTM-short and Glo-LTM-long.

### Antisense Transcription and Splice Variation

Antisense transcripts formed, together with lncRNA, a substantial part (12%) of the brain transcriptomes of *Cotesia*, and it is likely that a large proportion of the unknown transcripts are also non-coding. Non-coding RNA is known to be prevalent in neurons and particularly dendrites and synapses in other organisms as well, where it plays an important role in synaptic plasticity, learning and memory formation (Mercer et al., 2008; Earls et al., 2014; Smalheiser, 2014). Though unlike protein-coding transcripts, non-coding RNA tends to be not conserved across species (Smalheiser, 2014). Similarly, we observed only a small overlap in genes with antisense transcripts between two closely related *Cotesia* species, which confirms that the antisense transcript machinery has a strong species-specific background. We did, however, observe an overlap in GO terms related to signaling and transport that were enriched in the antisense transcripts. This suggests a common necessity of antisense transcription related to signaling and transport in the *Cotesia* brain.

Part of the genes to which an antisense transcript aligned did not have a sense transcript. It is possible that the antisense transcripts transcriptionally suppress these genes (Pelechano and Steinmetz, 2013). Many of these genes are mitochondrial genes, suggesting this type of transcription regulation is more specific to mitochondria.

Most of the antisense transcripts were either defined as antisense-to-protein or antisense-to-sense transcripts, not as both. Though this could be due to our stringent definitions of antisense transcripts, we showed that most of the antisense-to-sense transcripts we defined did not align to the protein-coding region of the sense transcripts but rather to the 3’-UTR and to a lesser extend to the 5’-UTR. Antisense transcripts that align to either 3’-UTR or the protein-coding region or the 5’-UTR could exert different effects on gene transcription and protein translation (Pelechano and Steinmetz, 2013).

The high incidence of splice variation in the brain transcriptomes of both *Cotesia* species and the increase in splice variation frequency in DE brain genes reveals that alternative splicing plays an important role in neuronal tissue and during memory formation. This is also seen in other organisms (Lipscombe, 2005). Splice variation in the *Cotesia* brain not only occurs at different time points after conditioning, but also between conditioning types in one species. Though the importance of non-coding RNA and splice variation has been recognized in brain functioning, the major challenge will be to pinpoint the role of individual non-coding RNA molecules and splice variants in learning and memory formation. This is particularly challenging for non-coding RNA due to its apparent species-specificity, which suggests either a quick evolution of non-coding RNA in the brain or that the majority of non-coding RNA in the brain is interchangeable, with a more general function than protein-coding RNA.

### Conclusion

In line with our hypothesis, we found many DE genes following conditioning. Our approach has resulted in the identification of several candidate genes that are potentially related to a single LTM type, whereas others were DE in two or three LTM-types. Moreover, we also identified genes that were expressed in opposite direction between wasps expressing either ARM or LTM, which may reflect ARM- or LTM-inducing (memory formation) or -inhibiting (memory forgetting) mechanisms. Some of the genes revealed by our study, *Olf186-F, nPKCδ, Rab40, Nep3, MEP-1* were not previously linked to learning and memory formation in insects.

Several genes we described displayed DE patterns in multiple memory types. However, most of the observed DE was unique to a single memory type, which may reflect strong intra- and interspecific variation in gene expression involved in LTM formation. Another source for this variation may lie in the limitations of the used method. Expression analysis using intact brain tissue reduces variation induced by the sampling procedure, but may also leave DE that is present in only small subsets of neurons undetected. Another caveat of the used methodology may be related to the used control. Instead of CS and US alone and backward pairing controls, which are often used for associative learning studies but not feasible for this conditioning paradigm, we made use of the Glo-ARM memory type as control treatment. Whereas this approach offered unique advantages, such as the detection of opposing gene expression patterns, it may also have introduced false negatives or positives, because the specific temporal dynamics between gene expression induced by conditioning, either or not related to LTM formation, may vary between single and spaced conditioning, between conditioning with different caterpillar species as US and between the two different wasp species. Substantial interspecific genetic variation was also observed in our previous study on LTM formation in the parasitic wasp genus *Nasonia* (Hoedjes et al., 2015). We consider the approach we used to identify gene expression related to LTM appropriate, but acknowledge that the specific role of the candidate genes generated by our study has to be further examined in follow up studies, involving quantitative PCR, RNAi and *in situ* hybridization. The current dataset will function as a useful resource for such studies, providing not only candidate genes, but also the complete brain transcriptomes covering sense and antisense transcripts.

A new finding from our study is the opposing gene expression patterns between ARM and LTM memory types. Since ARM does not require gene transcription, this opposing DE is remarkable. It suggests that inhibitory mechanisms may play an important role in the variation between ARM and LTM memory that we observe in *Cotesia* wasps. Possibly, the expression of ARM requires suppression of LTM in *Cotesia*, or* vice versa*. Examples of such opposing expression patterns were found in protein kinases (*nPKCδ*), regulators of alternative splicing (*Doa)*, mRNA translocation* (Tropomyosin)*, in small GTPases involved in regulation of synaptic plasiticty and membrane trafficking (*Rab40*) and epigenetic regulation (*histone* *demethylase 4B*). In addition we found that the *radish* gene, which is specifically involved in ARM formation in *Drosophila*, is downregulated in Glo-LTM.

This finding is in line with results on the classical model species in learning and memory research. The ubiquitous activity of memory suppressor mechanisms was already postulated by Abel et al. (1998), who posed the term memory suppressor genes for those genes that inhibit the formation or consolidation of memory. However, active memory degrading mechanisms exist as well, and the expression of genes involved in active forgetting have been described (Berry and Davis, 2014). Recently, a genetic screen with *D. melanogaster* revealed over 40 genes that enhance memory when inhibited by RNAi (Walkinshaw et al., 2015). Interestingly, in *D. melanogaster* it was shown that ARM and LTM are not independent memory forms that can occur in parallel, but are mutually exclusive (Isabel et al., 2004; Plaçais et al., 2012), because LTM formation requires ARM inhibition. Our own results show that in *C. glomerata* LTM and ARM may be mutually exclusive as well, because the formation of either ARM or LTM is specifically determined by the host species that is used as US (Kruidhof et al., 2012), whereas in *C. rubecula* ARM and LTM were reported to occur in parallel (Smid et al., 2007). The genes that are found in our study to be expressed in opposite direction are interesting candidates to be further studied in this context.

This is the first study to reveal the genetic background of natural variation in LTM formation within a single species and at the same time between closely related species. From our results, we conclude that the brain transcriptome analysis generated many candidate genes that are potentially involved in the observed natural variation in memory formation within and between both *Cotesia* species. The overlap in DE genes from our study with that of others reflects the conserved genetics of memory formation in the Animal Kingdom (Dubnau et al., 2003), which makes this study of fundamental interest for memory formation in general. Moreover, this work is of interest because of the importance of *Cotesia* wasps in crop protection and ecosystem functioning. The ability to learn is an important trait influencing efficiency of natural enemies, and a potential target to improve their performance (Kruidhof et al., 2014; Giunti et al., in press). As shown by our study, the rapid advances in high-throughput methodology enable the genetic analysis of ecologically relevant variation in behavioral traits in non-model animal species.

## Author Contributions

JV, LV and HS contributed to the conception and design of the work; JV, HG and ES contributed to the analysis of the data. JV, KH and HS contributed to the interpretation of the data. JV and HS contributed to the drafting of the work. JV, KH, HG, ES, LV and HS contributed to the revising of the manuscript, approve the final version to be published and agree to be accountable for all aspects of the work in ensuring that questions related to the accuracy or integrity of any part of the work are appropriately investigated and resolved.

## Conflict of Interest Statement

The authors declare that the research was conducted in the absence of any commercial or financial relationships that could be construed as a potential conflict of interest.
