# Supplementary material for: Differentially expressed genes linked to natural variation in long-term memory formation in Cotesia parasitic wasps
Source: Front Behav Neurosci. 2015 Sep 25;9:255. doi: 10.3389/fnbeh.2015.00255 (PMC4617343; doi:10.3389/fnbeh.2015.00255)
Supplement: Supplementary file 1 [file DataSheet_1.zip › Supplementary_Material/Supplementary Material.DOCX]

***Supplementary Material***

**Comparative transcriptomics of natural variation in long-term memory formation in *Cotesia* parasitic wasps**

**Joke JFA van Vugt^1,4*^, Katja M Hoedjes^2,5^, Henri C. van de Geest^3^, Elio WGM Schijlen^3^, Louise EM Vet^1,2^, Hans M Smid^2^**

^1^Department of Terrestrial Ecology, Netherlands Institute of Ecology (NIOO-KNAW), Wageningen, The Netherlands

^2^Laboratory of Entomology, Wageningen University, Wageningen, The Netherlands

^3^Applied Bioinformatics, Plant Research International, Wageningen, The Netherlands

^4^Current address: Department of Neurology, Rudolf Magnus Brain Centre, University Medical Center, Utrecht, The Netherlands

^5^Current address: Department of Ecology and Evolution, University of Lausanne, Lausanne, Switzerland

*** Correspondence:** Joke JFA van Vugt, Department of Neurology, Rudolf Magnus Brain Centre, University Medical Center, Heidelberglaan 100, Utrecht, The Netherlands.

j.f.a.vanvugt-2@umcutrecht.nl

1. **Supplementary Tables**

Supplementary Table 1. The number and average length of the different types of transcripts in the transcriptomes of *C. glomerata* and *C. rubecula*.

|  | ***C. glomerata*** | | | ***C. rubecula*** | | |
| --- | --- | --- | --- | --- | --- | --- |
|  | **#** | **%** | **length (bp)** | **#** | **%** | **length (bp)** |
| protein-coding (sense) | 25218 | 61,2 | 2785 | 24051 | 61,9 | 3003 |
| antisense | 3351 | 8,1 |  | 3040 | 7,8 |  |
| with a hit to a protein | 2636 | 6,4 | 1223 | 2308 | 5,9 | 1283 |
| with a hit to a sense transcript | 1096 | 2,7 | 952 | 1149 | 3,0 | 930 |
| with a hit to both | 381 | 0,9 |  | 417 | 1,1 |  |
| long non-coding RNA | 1619 | 3,9 | 737 | 2079 | 5,4 | 755 |
| with a putative ORF | 60 | 0,1 |  | 73 | 0,2 |  |
| unknown | 10994 | 26,7 | 824 | 9675 | 24,9 | 899 |
| with a putative ORF | 686 | 1,7 |  | 756 | 1,9 |  |
| Total | 41182 |  | 2044 | 38845 |  | 2213 |

Supplementary Table 2. The number and average length of the different types of differentially expressed (DE) transcripts in the transcriptomes of *C. glomerata* and *C. rubecula*.

|  | ***C. glomerata*** | | | ***C. rubecula*** | | |
| --- | --- | --- | --- | --- | --- | --- |
|  | **#** | **%** | **length (bp)** | **#** | **%** | **length (bp)** |
| protein-coding (sense) | 1168 | 61,5 | 2286 | 901 | 67,8 | 3132 |
| antisense | 145 | 7,6 |  | 94 | 7,1 |  |
| with a hit to a protein | 111 | 5,8 | 1398 | 71 | 5,3 | 1286 |
| with a hit to a sense transcript | 48 | 2,5 | 958 | 34 | 2,6 | 615 |
| with a hit to both | 14 | 0,7 |  | 11 | 0,8 |  |
| long non-coding RNA | 71 | 3,7 | 509 | 70 | 5,3 | 628 |
| with a putative ORF | 1 | 0,1 |  | 3 | 0,2 |  |
| unknown | 516 | 27,2 | 647 | 263 | 19,8 | 776 |
| with a putative ORF | 29 | 1,5 |  | 18 | 1,4 |  |
| Total | 1900 |  | 1689 | 1328 |  | 2390 |

Supplementary Table 3. The number and percentage of all sense, DE sense, all antisense and DE antisense genes in Glo-LTM-short, Glo-LTM-long and Rub-LTM-long.

|  | **All sense** | | **DE sense** | | **All antisense** | | **DE antisense** | |
| --- | --- | --- | --- | --- | --- | --- | --- | --- |
| Glo-LTM-short only | 23 | 0,2% | 104 | 10,4% | 41 | 2,3% | 16 | 12,4% |
| Glo-LTM-long only | 51 | 0,5% | 305 | 30,4% | 69 | 3,8% | 31 | 24,0% |
| Rub-LTM-long only | 952 | 9,0% | 495 | 49,4% | 800 | 44,3% | 79 | 61,2% |
| Glo-LTM-short & Glo-LTM-long | 1269 | 12,1% | 39 | 3,9% | 532 | 29,5% | 2 | 1,6% |
| Glo-LTM-short & Rub-LTM-long | 32 | 0,3% | 14 | 1,4% | 22 | 1,2% | 0 | 0,0% |
| Glo-LTM-long & Rub-LTM-long | 56 | 0,5% | 37 | 3,7% | 15 | 0,8% | 1 | 0,8% |
| Glo-LTM-short, Glo-LTM-long, Rub-LTM-long | 8146 | 77,4% | 8 | 0,8% | 327 | 18,1% | 0 | 0,0% |
| Total number of genes | 10529 |  | 1002 |  | 1806 |  | 129 |  |

Supplementary Table 4. The number and percentages of genes with a sense transcript, antisense-to-protein (a-to-p) transcript and antisense-to-sense (a-to-s) transcript in *C. glomerata* and *C. rubecula*.

|  | ***C. glomerata*** | | | ***C. rubecula*** | | |
| --- | --- | --- | --- | --- | --- | --- |
|  | **#** | **% sense** | **% antisense** | **#** | **% sense** | **% antisense** |
| sense only | 8700 | 90,6% |  | 8204 | 89,3% |  |
| a-to-p only | 152 |  | 14,4% | 182 |  | 15,6% |
| a-to-s only | 0 |  | 0,0% | 0 |  | 0,0% |
| sense & a-to-p | 146 | 1,5% | 13,8% | 172 | 1,9% | 14,8% |
| sense & a-to-s | 605 | 6,3% | 57,1% | 665 | 7,2% | 57,1% |
| a-to-p & a-to-s | 0 |  | 0,0% | 0 |  | 0,0% |
| sense, a-to-p & a-to-s | 156 | 1,6% | 14,7% | 145 | 1,6% | 12,5% |
| Total sense | 9607 |  |  | 9186 |  |  |
| Total antisense | 1059 |  |  | 1164 |  |  |

Supplementary Table 5. The number and percentages of the antisense-to-sense transcripts that aligned to (part of) the 5’-UTR region, (part of) the protein-coding region (coding) and (part of) the 3’-UTR region in *C. glomerata* and *C. rubecula* of sense transcripts that align to *N. vitripennis* proteins with more than 90% protein coverage. Only the antisense-to-sense transcripts that align to the 5’-UTR, protein-coding region and 3’-UTR, align to the full protein-coding region.

|  | ***C. glomerata*** | | ***C. rubecula*** | | |
| --- | --- | --- | --- | --- | --- |
|  | **#** | **%** | **#** | **%** |  |
| 5'-UTR only | 79 | 11,5% | 79 | 11,6% |  |
| coding only | 97 | 14,2% | 75 | 11,0% |  |
| 3'-UTR only | 168 | 24,5% | 195 | 28,6% |  |
| 5'-UTR & coding | 67 | 9,8% | 54 | 7,9% |  |
| 5'-UTR & 3'-UTR | 0 | 0,0% | 0 | 0,0% |  |
| coding & 3'-UTR | 188 | 27,4% | 180 | 26,4% |  |
| 5'-UTR, coding & 3'-UTR | 86 | 12,6% | 98 | 14,4% |  |
| Total | 685 |  | 681 |  |  |

Supplementary Table 6. The 176 annotated genes with antisense-to-sense (a-to-s) and antisense-to-protein (a-to-p) transcripts for *C. glomerata* (Glo) and *C. rubecula* (Rub). The conditioning type and time point of the DE antisense transcripts (bold) are also indicated.

Supplementary Table 7. Numbers and percentages of the up- and downregulated transcripts at each indicated time point in Glo-LTM-short and Glo-LTM-long.

|  | **Glo-LTM-short** | | **Glo-LTM-long** | |
| --- | --- | --- | --- | --- |
|  | **DE (up)** |  | **DE (up)** |  |
| 15m only | 72 (36) | 15,3% | 456 (219) | 46,0% |
| 1h only | 235 (90) | 49,9% | 176 (79) | 17,8% |
| 4h only | 81 (37) | 17,2% | 172 (87) | 17,4% |
| 15m & 1h | 31 (16) | 6,6% | 42 (30) | 4,2% |
| 15m & 4h | 9 (3) | 1,9% | 56 (40) | 5,7% |
| 1h & 4h | 15 (8) | 3,2% | 37 (28) | 3,7% |
| 15m, 1h & 4h | 28 (12) | 5,9% | 52 (46) | 5,2% |
| Total | 471 (202) |  | 991 (529) |  |

Supplementary Table 8. Numbers and percentages of the up- and downregulated transcripts at each indicated time point in Rub-LTM-long.

|  | **Rub-LTM-long** | |
| --- | --- | --- |
|  | **DE (up)** |  |
| 1h only | 223 (92) | 16,6% |
| 4h only | 298 (131) | 22,2% |
| 24h only | 515 (216) | 38,4% |
| 1h & 4h | 91 (49) | 6,8% |
| 1h & 24h | 64 (33) | 4,8% |
| 4h & 24h | 74 (43) | 5,5% |
| 1h, 4h & 24h | 76 (61) | 5,7% |
| Total | 1341 (625) |  |

Supplementary Table 9. The number of genes and DE genes with multiple transcripts.

|  | ***C. glomerata*** | **%** | ***C. rubecula*** | **%** |
| --- | --- | --- | --- | --- |
| Genes | 23.287 |  | 21.946 |  |
| with multiple transcripts | 5.692 | 24 | 5.178 | 24 |
| Transcripts | 41.182 |  | 38.845 |  |
| of genes with multiple transcripts | 23.587 | 57 | 22.077 | 57 |
| DE genes | 1.570 |  | 1.075 |  |
| with multiple transcripts | 1.122 | 71 | 725 | 67 |
| with multiple DE transcripts | 232 | 15 | 160 | 15 |
| with different DE transcripts at multiple memory types | 168 | 11 |  |  |
| DE transcripts | 1.900 |  | 1.328 |  |
| of genes with multiple transcripts | 1.452 | 76 | 979 | 74 |
| of genes with multiple DE transcripts | 562 | 30 | 413 | 31 |
| of genes with different DE transcripts at multiple memory types | 600 | 32 |  |  |

Supplementary Table 10. Genes with different splice variants up- or downregulated in different conditioning types, together with their annotated gene name, *Drosophila* gene ID, and the splice variants that were DE at each time point and in each conditioning type.

Supplementary Table 11. Genes of whom all DE transcripts had an opposing expression pattern between LTM and ARM, with their annotated gene name, *Drosophila* gene ID, and the splice variants that were DE at each time point and in each conditioning type. Genes are clustered according to the memory type in which they were DE, and alternative splice forms are clustered together. Note that opposing expression patterns were recognized either within the same splice form or across different splice forms, and that the factor time after conditioning was not taken into account, given the variation in consolidation speed, and in the duration between single and spaced conditioning.

Supplementary Table 12. All protein-coding genes DE in the *Cotesia* LTM conditioning types with their annotated gene name, *Drosophila* gene ID, and the splice variants that were DE at each time point and in each conditioning type.

Supplementary Table 13. The 79 genes involved in memory formation from literature, together with their *Drosophila* gene ID and expression in each conditioning type.

Supplementary Table 14. GO terms, each with 10 or more genes, of the categories biological process and molecular function that were enriched in any of the LTM conditioning types in up- and downregulated transcripts of each time point after conditioning. GO terms enriched with P < 0.001 are indicated in black, with 0.01 < P < 0.001 in dark grey, 0.05 < P < 0.01 in light grey.

Supplementary Table 15. The 106 DE genes underlying the 8 enriched GO terms described in Figure 4 with their gene name, expression pattern, *Cotesia* gene ID and *Drosophila* gene ID.

Supplementary Table 16. The 37 DE genes underlying the enriched GO terms ‘Neurological system process’ and ‘Cytoskeletal protein binding’ described in Supplementary Table 14 with their gene name, expression pattern, *Cotesia* gene ID and *Drosophila* gene ID.

Supplementary Table 17. Genes with DE antisense transcripts, classified as antisense-to-protein (a-to-p) and antisense-to-sense (a-to-s), at each of the time points after conditioning for all 3 LTM conditioning types. The sense transcripts to which these antisense transcripts aligned and whether these were DE are also provided, as well as the *Drosophila* gene ID. Gene names starting with “Potential” were annotated using blastn (e-value 1E-10) against the NCBI nt database, without verification of a reciprocal blast.

1. **Supplementary Figures**

Supplementary Figure 1. Expression plots for each time point of each conditioning type after removing 3880 transcripts with a *Burkholderia* blast hit, where the log_2_-counts-per-million (logCPM) is plotted against the log_2_-fold-change (logFC) compared to transcripts of unconditioned wasps. Each dot represents one transcript. Red dots indicate DE transcripts. The blue line indicates a 2-fold change compared to expression levels of transcripts of unconditioned wasps. We did not remove all transcripts with a bacterial blast hit, because the blast was not performed reciprocal and therefore we could not be sure that these transcripts were of bacterial origin. The approximate 100 transcripts with a bacterial hit that were left after removing all transcripts with a hit to *Burkholderia* in *C. glomerata*, were considered too little to distort the differential expression analysis and structural annotation statistics of the transcriptome. Transcripts of bacterial origin were not considered in the GO term enrichment analysis, because they were not functionally annotated.

Supplementary Figure 2. Multi-dimension scaling plots of Glo-LTM-short (A), Glo-LTM-long (B), Glo-ARM (C) and Rub-LTM-long (D), where the numbers at the end of each data point corresponds to the replicate number and “No” corresponds to the unconditioned samples. The grouping of the samples per replicate, rather than by sample type, for *C. glomerata* and to a lesser extend for *C. rubecula* reveals the replicate effect.

1. **Supplementary References**

Akalal D.B., Yu D., and Davis R.L. (2011). The long-term memory trace formed in the Drosophila α/β mushroom body neurons is abolished in long-term memory mutants. *J. Neurosci.* 31, 5643-5647. doi: 10.1523/JNEUROSCI.3190-10.2011

Alberini C.M. (2009). Transcription factors in long-term memory and synaptic plasticity. *Physiol. Rev.* 89, 121-145. doi: 10.1152/physrev.00017.2008

Banerjee P., Schoenfeld B.P., Bell A.J., Choi C.H., Bradley M.P., Hinchey P., et al. (2010). Short- and Long-Term Memory Are Modulated by Multiple Isoforms of the Fragile X Mental Retardation Protein. *J. Neurosci.* 30, 6782-6792.

Bayliss A., Roselli G., and Evans P.D. (2013). A comparison of the signalling properties of two tyramine receptors from Drosophila. *J. Neurochem.* 125, 37-48. doi: 10.1111/jnc.12158

Bettencourt da Cruz A., Wentzell J., and Kretzschmar D. (2008). Swiss Cheese, a protein involved in progressive neurodegeneration, acts as a noncanonical regulatory subunit for PKA-C3. *J. Neurosci.* 28, 10885-10892. doi: 10.1523/JNEUROSCI.3015-08.2008

Bevilaqua L.R., Medina J.H., Izquierdo I., and Cammarota M. (2005). Memory consolidation induces N-methyl-D-aspartic acid-receptor- and Ca2+/calmodulin-dependent protein kinase II-dependent modifications in alpha-amino-3-hydroxy-5-methylisoxazole-4-propionic acid receptor properties. *Neuroscience.* 136, 397-403. doi: 10.1016/j.neuroscience.2005.08.007

Burke C.J., Huetteroth W., Owald D., Perisse E., Krashes M.J., Das G., et al. (2012). Layered reward signalling through octopamine and dopamine in Drosophila. *Nature.* 492, 433-437. doi: doi:10.1038/nature11614

Chatwin H.M., Rudling J.E., Patel D., Reale V., and Evans P.D. (2003). Site-directed mutagenesis studies on the Drosophila octopamine/tyramine receptor. *Insect. biochemistry. and. molecular. biology.* 33, 173-184. doi: 10.1016/S0965-1748(02)00188-1

Chiba A., Snow P., Keshishian H., and Hotta Y. (1995). Fasciclin III as a synaptic target recognition molecule in Drosophila.

Copf T., Goguel V., Lampin-Saint-Amaux A., Scaplehorn N., and Preat T. (2011). Cytokine signaling through the JAK/STAT pathway is required for long-term memory in Drosophila. *Proc. Natl. Acad. Sci. U. S. A.* 108, 8059-8064. doi: 10.1073/pnas.1012919108

Copf T., Goguel V., Lampin-Saint-Amaux A., Scaplehorn N., and Preat T. (2011). Cytokine signaling through the JAK/STAT pathway is required for long-term memory in Drosophila. *Proc. Natl. Acad. Sci. U. S. A.* 108, 8059-8064. doi: 10.1073/pnas.1012919108

Eickhoff R., and Bicker G. (2012). Developmental expression of cell recognition molecules in the mushroom body and antennal lobe of the locust Locusta migratoria. *J. Comp. Neurol.* 520, 2021-2040. doi: 10.1002/cne.23026

Gallagher H.C., Murphy K.J., Foley A.G., and Regan C.M. (2001). Protein kinase C delta regulates neural cell adhesion molecule polysialylation state in the rat brain. *J. Neurochem.* 77, 425-434.

Gillespie J.M., and Hodge J.J. (2013). CASK regulates CaMKII autophosphorylation in neuronal growth, calcium signaling, and learning. *Front. Mol. Neurosci.* 6:27 doi: 10.3389/fnmol.2013.00027

Hearn M.G., Ren Y., McBride E.W., Reveillaud I., Beinborn M., and Kopin A.S. (2002). A Drosophila dopamine 2-like receptor: Molecular characterization and identification of multiple alternatively spliced variants. *Proc. Natl. Acad. Sci. U. S. A.* 99, 14554-14559. doi: 10.1073/pnas.202498299

Hirano Y., Masuda T., Naganos S., Matsuno M., Ueno K., Miyashita T., et al. (2013). Fasting launches CRTC to facilitate long-term memory formation in Drosophila. *Science.* 339, 443-446. doi: 10.1126/science.1227170

Ishimoto H., Wang Z., Rao Y., Wu C.F., and Kitamoto T. (2013). A novel role for ecdysone in Drosophila conditioned behavior: linking GPCR-mediated non-canonical steroid action to cAMP signaling in the adult brain. *PLoS. Genet.* 9:e1003843 doi: 10.1371/journal.pgen.1003843

Iyer S.C., Wang D., Iyer E.P., Trunnell S.A., Meduri R., Shinwari R., et al. (2012). The RhoGEF trio functions in sculpting class specific dendrite morphogenesis in Drosophila sensory neurons. *PLoS. One.* 7:e33634 doi: 10.1371/journal.pone.0033634

Johnson O., Becnel J., and Nichols C.D. (2011). Serotonin receptor activity is necessary for olfactory learning and memory in Drosophila melanogaster. *Neuroscience.* 192, 372-381. doi: 10.1016/j.neuroscience.2011.06.058

Keene A.C., and Waddell S. (2007). Drosophila olfactory memory: single genes to complex neural circuits. *Nat. Rev. Neurosci.* 8, 341-354. doi: 10.1038/nrn2098

Mery F., Belay A.T., So A.K.C., Sokolowski M.B., and Kawecki T.J. (2007). Natural polymorphism affecting learning and memory in Drosophila. *Proc. Natl. Acad. Sci. U. S. A.* 104, 13051-13055.

Ohhara Y., Kayashima Y., Hayashi Y., Kobayashi S., and Yamakawa-Kobayashi K. (2012). Expression of β-adrenergic-like octopamine receptors during Drosophila development. *Zoolog Sci.* 29(2), 83-89. doi: 10.2108/zsj.29.83.

Pagani M.R., Oishi K., Gelb B.D., and Zhong Y. (2009). The phosphatase SHP2 regulates the spacing effect for long-term memory induction. *Cell.* 139, 186-198. doi: 10.1016/j.cell.2009.08.033

Pai T.P., Chen C.C., Lin H.H., Chin A.L., Lai J.S., Lee P.T., et al. (2013). Drosophila ORB protein in two mushroom body output neurons is necessary for long-term memory formation. *Proc. Natl. Acad. Sci. U. S. A.* 110, 7898-7903. doi: 10.1073/pnas.1216336110

Schmitt J.M., Guire E.S., Saneyoshi T., and Soderling T.R. (2005). Calmodulin-dependent kinase kinase/calmodulin kinase I activity gates extracellular-regulated kinase-dependent long-term potentiation. *The. Journal. of. neuroscience.* 25, 1281-1290.

Schwaerzel M., Jaeckel A., and Mueller U. (2007). Signaling at A-kinase anchoring proteins organizes anesthesia-sensitive memory in Drosophila. *J. Neurosci.* 27, 1229-1233.

Sharma S., Darland D., Lei S., Rakoczy S., and Brown-Borg H.M. (2012). NMDA and kainate receptor expression, long-term potentiation, and neurogenesis in the hippocampus of long-lived Ames dwarf mice. *Age (Dordr.)* 34(3), 609-620. doi: 10.1007/s11357-011-9253-1.

Shuai Y., Lu B., Hu Y., Wang L., Sun K., and Zhong Y. (2010). Forgetting Is Regulated through Rac Activity in Drosophila. *Cell.* 140, 579-589. doi: 10.1016/j.cell.2009.12.044

Song H., Sun Y., Zhang Y., and Li M. (2009). Molecular cloning and characterization of Bombyx mori CREB gene. *Arch. Insect. Biochem. Physiol.* 71, 31-44. doi: 10.1002/arch.20292

Tejedor F., Zhu X.R., Kaltenbach E., Ackermann A., Baumann A., Canal I., et al. (1995). minibrain: a new protein kinase family involved in postembryonic neurogenesis in Drosophila. *Neuron.* 14, 287-301.

Thiels E., Kanterewicz B.I., Knapp L.T., Barrionuevo G., and Klann E. (2000). Protein phosphatase-mediated regulation of protein kinase C during long-term depression in the adult hippocampus in vivo. *J. Neurosci.* 20, 7199-7207.

Umemori J., Takao K., Koshimizu H., Hattori S., Furuse T., Wakana S., and Miyakawa T. (2013). ENU-mutagenesis mice with a non-synonymous mutation in Grin1 exhibit abnormal anxiety-like behaviors, impaired fear memory, and decreased acoustic startle response. *BMC Res. Notes.* 6-203. doi: 10.1186/1756-0500-6-203.

Yamamoto S., and Seto E.S. (2014). Dopamine Dynamics and Signaling in Drosophila: An Overview of Genes, Drugs and Behavioral Paradigms. *Experimental. Animals.* 63, 107-119. doi: /10.1538/expanim.63.107

Zisopoulou S., Asimaki O., Leondaritis G., Vasilaki A., Sakellaridis N., Pitsikas N., et al. (2013). PKC-epsilon activation is required for recognition memory in the rat. *Behav. Brain. Res.* 253, 280-289. doi: 10.1016/j.bbr.2013.07.036
