# Supplementary figures and images for: Differentially expressed genes linked to natural variation in long-term memory formation in Cotesia parasitic wasps
Source: Front Behav Neurosci. 2015 Sep 25;9:255. doi: 10.3389/fnbeh.2015.00255 (PMC4617343; doi:10.3389/fnbeh.2015.00255)

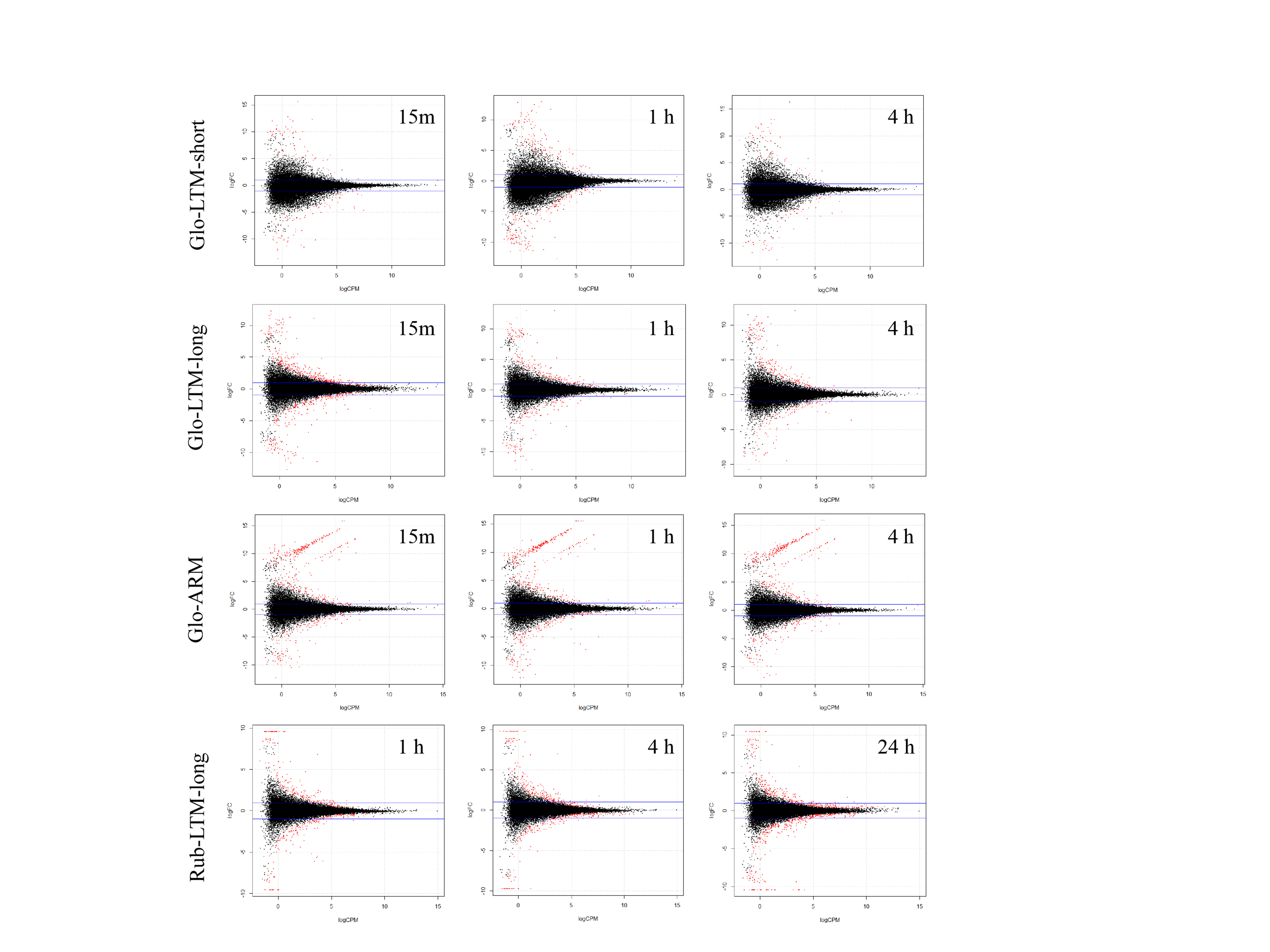

Supplement: Supplementary file 1 [file DataSheet_1.zip › Supplementary_Material/Supplementary Figure 1.TIF]

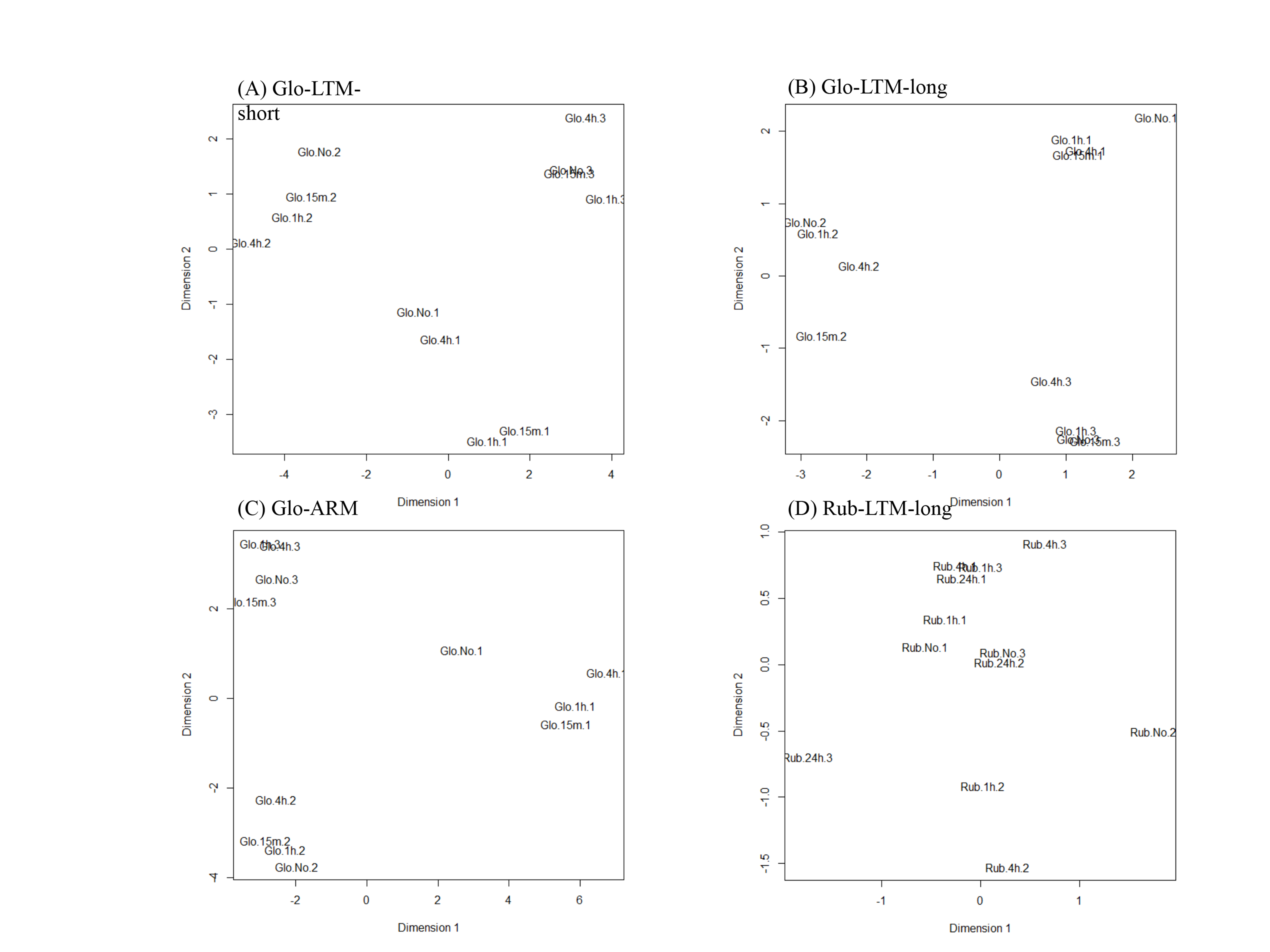

Supplement: Supplementary file 1 [file DataSheet_1.zip › Supplementary_Material/Supplementary Figure 2.TIF]
